# Discovery and characterization of Isofistularin-3, a marine brominated alkaloid, as a new DNA demethylating agent inducing cell cycle arrest and sensitization to TRAIL in cancer cells

**DOI:** 10.18632/oncotarget.8210

**Published:** 2016-03-19

**Authors:** Cristina Florean, Michael Schnekenburger, Jin-Young Lee, Kyung Rok Kim, Aloran Mazumder, Sungmi Song, Jae-Myun Kim, Cindy Grandjenette, Jeoung-Gyun Kim, Ah-Young Yoon, Mario Dicato, Kyu-Won Kim, Christo Christov, Byung-Woo Han, Peter Proksch, Marc Diederich

**Affiliations:** ^1^ Laboratoire de Biologie Moléculaire et Cellulaire du Cancer, Hôpital Kirchberg, Lëtzebuerg, Luxembourg; ^2^ Department of Pharmacy, Research Institute of Pharmaceutical Sciences, College of Pharmacy, Seoul National University, Gwanak-gu, Korea; ^3^ SNU-Harvard Neurovascular Protection Center, College of Pharmacy and Research Institute of Pharmaceutical Sciences, Seoul National University, Gwanak-gu, Korea; ^4^ Faculté de Médecine, Université de Lorraine, Nancy, France; ^5^ Institut für Pharmazeutische Biologie und Biotechnologie, Heinrich-Heine-Universität Düsseldorf, Düsseldorf, Germany

**Keywords:** leukemia, DNMT inhibitor, TSG hypermethylation, cell cycle arrest, autophagy

## Abstract

We characterized the brominated alkaloid Isofistularin-3 (Iso-3), from the marine sponge *Aplysina aerophoba*, as a new DNA methyltransferase (DNMT)1 inhibitor. Docking analysis confirmed our *in vitro* DNMT inhibition data and revealed binding of Iso-3 within the DNA binding site of DNMT1. Subsequent increased expression of tumor suppressor gene aryl hydrocarbon receptor (AHR) could be correlated to decreased methylation of CpG sites within the essential Sp1 regulatory region of its promoter. Iso-3 induced growth arrest of cancer cells in G0/G1 concomitant with increased p21 and p27 expression and reduced cyclin E1, PCNA and c-myc levels. Reduced proliferation was accompanied by morphological changes typical of autophagy revealed by fluorescent and transmission electron microscopy and validated by LC3I-II conversion. Furthermore, Iso-3 strongly synergized with tumor-necrosis-factor related apoptosis inducing ligand (TRAIL) in RAJI [combination index (CI) = 0.22] and U-937 cells (CI = 0.21) and increased TRAIL-induced apoptosis *via* a mechanism involving reduction of survivin expression but not of Bcl-2 family proteins nor X-linked inhibitor of apoptosis protein (XIAP). Iso-3 treatment decreased FLIP_L_ expression and triggered activation of endoplasmatic reticulum (ER) stress with increased GRP78 expression, eventually inducing TRAIL receptor death receptor (DR)5 surface expression. Importantly, as a potential candidate for further anticancer drug development, Iso-3 reduced the viability, colony and *in vivo* tumor forming potential without affecting the viability of PBMCs from healthy donors or zebrafish development.

## INTRODUCTION

Epigenetic mechanisms, including DNA methylation and histone modifications, play a central role in all physiological cellular functions, and their de-regulation is a well-established feature of cancer cells [1]. The possibility to revert tumor suppressor gene (TSG) hypermethylation, hence re-establishing TSG functions, represents a major perspective in anticancer therapy. Two demethylating agents, the nucleoside analogs 5-azacytidine and 5-aza-2′-deoxycytidine (DAC) have been approved for the treatment of myelodysplastic syndrome (MDS) [2–5]. However, their toxic effects in the clinical context triggered research of novel non-nucleoside molecules with demethylating activity and structurally different compounds have been reported so far to affect DNMT activity *in vitro* and TSG methylation in cell models [2, 6–8].

The chemical versatility of marine organisms has made them one of the most powerful sources of molecules for biomedical use. Marine sponges are a very rich source of natural secondary metabolites, many of which demonstrated interesting anticancer activities. Cytarabine (Ara-C) was the first sponge-derived compound to reach clinical use against leukemia, and constitutes now one of the standard treatments for hematological diseases [9–11].

**Figure 1:**
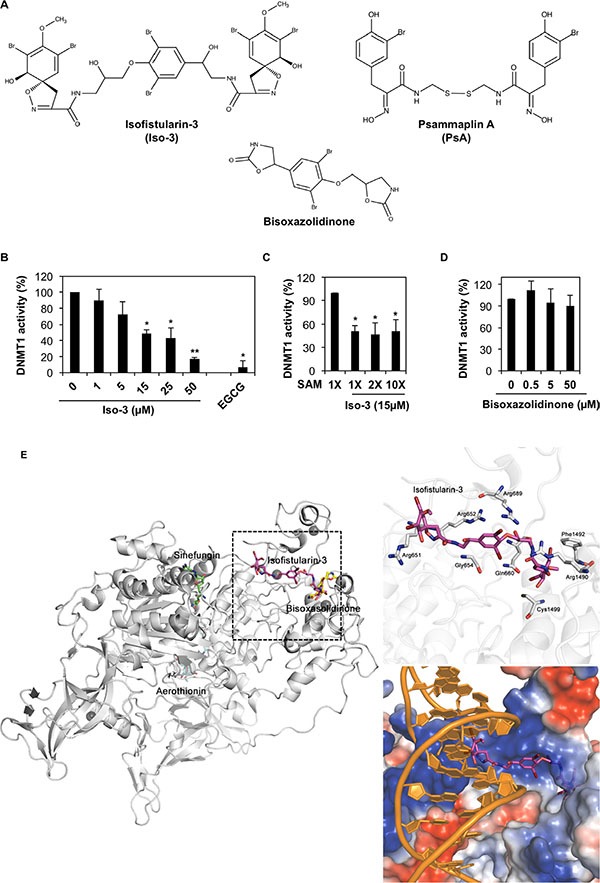
Iso-3 is a new DNMT1 inhibitor interacting with the DNA binding site of DNMT1 (**A**) Chemical structures of Iso-3, Psammaplin A and bisoxasolidinone. (**B**) *In vitro* activity of purified DNMT1 was tested in the presence of increasing concentrations of Iso-3. Data are reported as percentage of DNMT1 activity respect to the control. (**C**) Iso-3 inhibitory activity against DNMT1 was measured in the presence of increasing concentrations of SAM. (**D**) *In vitro* activity of purified DNMT1 in the presence of increasing concentrations of bisoxasolidinone. Histograms represent the mean ± SD of three independent experiments. (**E**) Docking poses of Iso-3, aerothionin, and bisoxasolidinone on the crystal structure of DNMT1 (PDB-Code: 3SWR). DNMT1 protein is represented as cartoon and stick models with carbon, nitrogen, oxygen, and sulphur in white, blue, red, and yellow, respectively. Sinefungin, Iso-3, aerothionin, and bisoxasolidinone are shown as stick models with carbon colored in green, magenta, cyan, and yellow; nitrogen, oxygen, and bromide atoms colored in blue, red, and brown, respectively. Double-stranded DNA model was adopted from the crystal structure of DNMT1-DNA complex (PDB-Code: 3PTA) and colored in orange. Electrostatic potential surface of DNMT1 was calculated and represented as negatively and positively charged surfaces in red and blue shade, respectively.

Brominated compounds may present interesting epigenetic modulatory potential. The sponge-derived bromotyrosine derivative psammaplin-A (PsA, Figure 1A) and its derivatives were previously shown to inhibit both methyltransferase and histone deacetylase (HDAC) activities [12–16]. Isofistularin-3 (Iso-3, Figure 1A), a brominated alkaloid derived from *Aplysina aerophoba*, a marine sponge from the Mediterranean sea, was selected here due to its structural resemblance to PsA, being both bromotyrosine derivatives. No mechanistic study has been conducted so far to investigate the anticancer activity of this molecule. Nevertheless, cytotoxic activity against HeLa cells has been reported for Iso-3 [17], and a cytostatic effect was reported for Iso-3 structural isomer fistularin-3, against hematological cancer lines [18], indicating potential benefits in the use of Iso-3 against cancer.

As outcome of the induced gene expression modulation, epigenetic agents are known to produce a variety of cellular effects, ranging from cell cycle arrest and autophagy to cell death [1, 19, 20]. All these features contribute to the increasingly high interest raised by these molecules in anticancer research.

In this study, we describe Iso-3 as a new DNMT1 inhibitor with a strong impact on cancer cell proliferation, the induction of autophagy and a promising synergistic chemosensitizing activity to tumor-necrosis-factor related apoptosis inducing ligand (TRAIL) in combination treatments.

## RESULTS

### Isofistularin-3 inhibits DNMT1 *in vitro* by binding to the DNA interacting pocket of the enzyme

The ability of Iso-3 to reduce DNMT1 activity was determined by performing a molecular screening of a library of natural compounds, using a biochemical *in vitro* assay. Along with few other hits, (Supplementary Table S1 and Supplementary Figure S1A) we identified Iso-3 as a new DNMT1 inhibitor. The analysis revealed an inhibition of the purified enzyme by Iso-3 with an IC_50_ of 13.5 ± 5.4 μM. Green tea polyphenol EGCG was used as a positive control for *in vitro* DNMT1 inhibition. (Figure 1B). Addition of Triton X100 (0.01%) [21] did not affect the inhibitory activity of Iso-3 (Supplementary Figure S2A), arguing against a potential aggregation-based inhibition. Increasing the concentration of the *S*-adenosyl methionine (SAM) cofactor did not affect Iso-3 activity, supporting a mechanism of DNMT1 inhibition that differs from competition with SAM (Figure 1C). Interestingly, the Iso-3 derivative bisoxazolidinone (Figure 1A), devoid of brominated side arms, failed to inhibit DNMT1 activity (Figure 1D).

In order to gain insight into the interaction modes of Iso-3 and human DNMT1, we implemented docking simulation using PatchDock software [22, 23]. In parallel, the Iso-3 structural analog aerothionin, presenting lower DNMT1 inhibitory activity (Supplementary Table S1) as well as the inactive analog bisoxasolidinone, were analyzed. Before assessing the affinity between compounds and DNMT1, we performed a control docking experiment with sinefungin to the crystal structure of DNMT1 (Protein Data Bank ID: 3SWR) after sinefungin was removed from the complex structure. Predicted binding score of Iso-3 was 7,426, whereas that of sinefungin was 5,372 from the control docking experiment (RMSD distance from sinefungin in the crystal structure of DNMT1-sinefungin complex: 1.292 Å). Predicted docking scores for aerothionin and bisoxasolidinone were 6,414 and 4,292, respectively (Figure 1E).

In the docking analyses, Iso-3 was predicted to be located in a DNA-binding CXXC domain with more stable energy score than that of sinefungin, whereas sinefungin is bound to SAM site of DNMT1. Iso-3 appeared to interact with positively charged residues, which might affect DNA binding activity. Electrostatic surface view of the crystal structure of DNMT1 suggests that electrostatic interaction is a major interacting force between Iso-3 and DNMT1. When the results were compared with the crystal structure of DNMT1-DNA complex (Protein Data Bank ID: 3PTA) [24], Iso-3 seemed to be located in the DNA binding site (Figure 1E). However, bisoxasolidinone was predicted to be located between CXXC domain and target recognition domain on DNMT1 and it may not interrupt binding of DNA or any other ligands with DNMT1. The binding score of bisoxasolidinone was lower than that of sinefungin. From our DNMT1 activity tests and docking studies, we hypothesize that Iso-3 could inhibit the interaction between DNMT1 and DNA.

### Isofistularin-3 modifies AHR promoter methylation and increases AHR expression in RAJI cells

**Figure 2:**
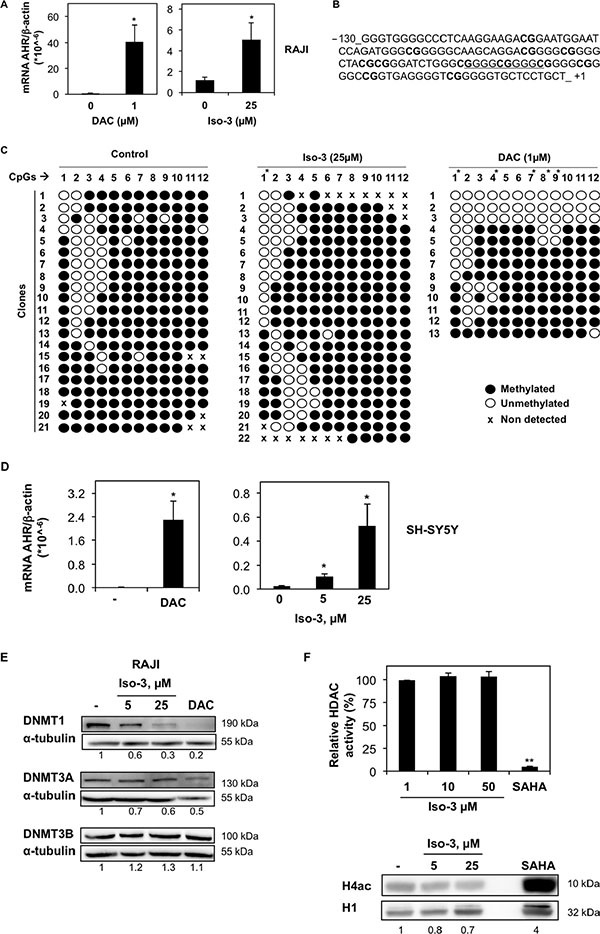
Iso-3 increases AHR expression and induces AHR promoter demethylation (**A**) mRNA levels of AHR in RAJI cells measured after 72 h of treatment with 1 μM DAC or 25 μM Iso-3. (**B**) DNA sequence of the AHR promoter region selected for methylation analysis. The underlined sequence corresponds to SNP rs71010234. (**C**) The methylation pattern of AHR promoter in RAJI cells was revealed upon 72 h of treatment with Iso-3 or DAC. Results show the methylation status for each of the 12 CpGs upstream of the transcription start site (horizontal numbering), in the different clones sequenced (vertical numbering). Open and closed circles indicate unmethylated and methylated CGs, respectively. (**D**) mRNA levels of AHR in SH-SY5Y cells measured after 72 h of treatment with 1 μM DAC or indicated Iso-3 concentrations. (**E**) Protein expression levels of DNMT isoforms in RAJI cells treated 72 h with Iso-3 at indicated doses or 1 μM DAC. (**F**) Top panel: *in vitro* total HDAC activity in presence of indicated Iso-3 doses or 2 μM SAHA. Data are reported as percentage of HDAC activity respect to the control. Bottom panel: acetylated histone 4 (H4ac) levels in Iso-3- or 2 μM SAHA-treated RAJI cells (24 h). Histone H1 (H1) was used as a loading control. All histograms represent the mean ± SD of three independent experiments. All blots are representative of three independent experiments.

To ascertain that DNA methylation was affected by Iso-3, consistently with inhibition of DNMT1 activity, we investigated expression levels of the aryl hydrocarbon receptor (AHR) gene in Burkitt's lymphoma RAJI cells. Several reports established a tumor suppressive activity of this gene [25–27]. Furthermore, hypermethylation and repression of AHR gene were previously reported in various leukemia cell models [28]. Preliminary work conducted by our group suggested that AHR promoter is methylated in RAJI cells; consistently, a strong increase in AHR mRNA expression was obtained by DAC treatment (Figure 2A).

We found that AHR mRNA expression level was 5.1 times higher after 72 h exposure to 25 μM Iso-3 (Figure 2A), a concentration causing a low viability decrease. Next, we performed methylation analysis of AHR promoter CpG island in RAJI cells. DNA was bisulfite-converted and the fragment of interest was cloned and sequenced. We focused on a 130-nt fragment above the transcription start site, containing 12 CG sites, which belong to the Sp1 regulatory region of AHR promoter (Figure 2B). Iso-3 treatment resulted in a CG-specific decrease in methylation levels (Figure 2C). The first CG site analyzed (−108) appeared to be the most affected, with 50% of demethylation with respect to the control sample (Figure 2C). A comparable result at this CG site was obtained with DAC, used as a positive control. In addition, the CGs in positions −88, −73 and −60 show a decrease of around 10% in their methylation levels after Iso-3 treatment. Intriguingly, the most affected CG site (−108) does not belong to a GC-box (Sp1 recognition element) but is located in a flanking position. This result confirms the importance of the methylation status of CG sites flanking the Sp1 consensus elements, in agreement with several previous reports [29–31].

To attempt to generalize our results, we analyzed AHR mRNA levels in neuroblastoma SH-SY5Y cells, another model lacking AHR expression [32]. DAC treatment increased substantially AHR mRNA level, suggesting for the first time that AHR silencing relies on DNA methylation in this neuroblastoma model. Moreover, treatment with Iso-3 resulted also in a significant AHR mRNA increase in this cell line, to an even stronger degree compared to RAJI cells (increase of 4.25 and 21.5 fold at 5 and 25 μM, respectively) (Figure 2D). However, we did not observe any increase in Iso-3-treated JURKAT T cells, another AHR-negative DAC-responsive cell line (data not shown).

We next analyzed protein levels of AHR in DAC- and Iso-3-treated RAJI cells. Despite the mRNA increase obtained with both drugs, we failed to detect AHR protein in any of the treated samples (Supplementary Figure S1B). These data suggest translational inhibition as a secondary mechanism of AHR repression in this cell line, independent of its epigenetic regulation.

Compounds showing DNMT inhibitory activity are often reported to affect DNMT protein levels [33–38]. By analyzing the expression levels of DNMT isoforms in RAJI cells treated with Iso-3 for 72 hours, we could observe a decrease of DNMT1, whereas other DNMT isoforms were not affected by such treatments (Figure 2E). By analyzing mRNA levels, we found no change in gene transcription that could justify a decrease of DNMT1 protein levels (Supplementary Figure S3A). This decrease appeared nevertheless to be specific for RAJI cells, as we did not detect it in other cell lines, whereas DAC treatment led to DNMT1 degradation in all cell models tested (Figure 2E and Supplementary Figure S3B). In the same context, we observed a decrease of DNMT3A in Iso-3-treated JURKAT and HL-60 cells, with appearance of a lower band in HL-60 cells, probably due to high cell death induction and proteolytic cleavage of the protein in this cell line. In the same cell line, DNMT1 protein levels appeared increased (Supplementary Figure S3B).

We further assessed the epigenetic potential of Iso-3 by analyzing its HDAC inhibitory activity. Our data show that Iso-3 has no effect on *in vitro* total HDAC activity (Figure 2F). Moreover, no increase but rather a decrease of the acetylated form of histone 4 (H4) was recorded in Iso-3-treated RAJI cells, in comparison with the reference pan-HDAC inhibitor suberoylanilide hydroxamic acid (SAHA).

### Isofistularin-3 arrests cancer cells in G0/G1 cell cycle phase

**Figure 3:**
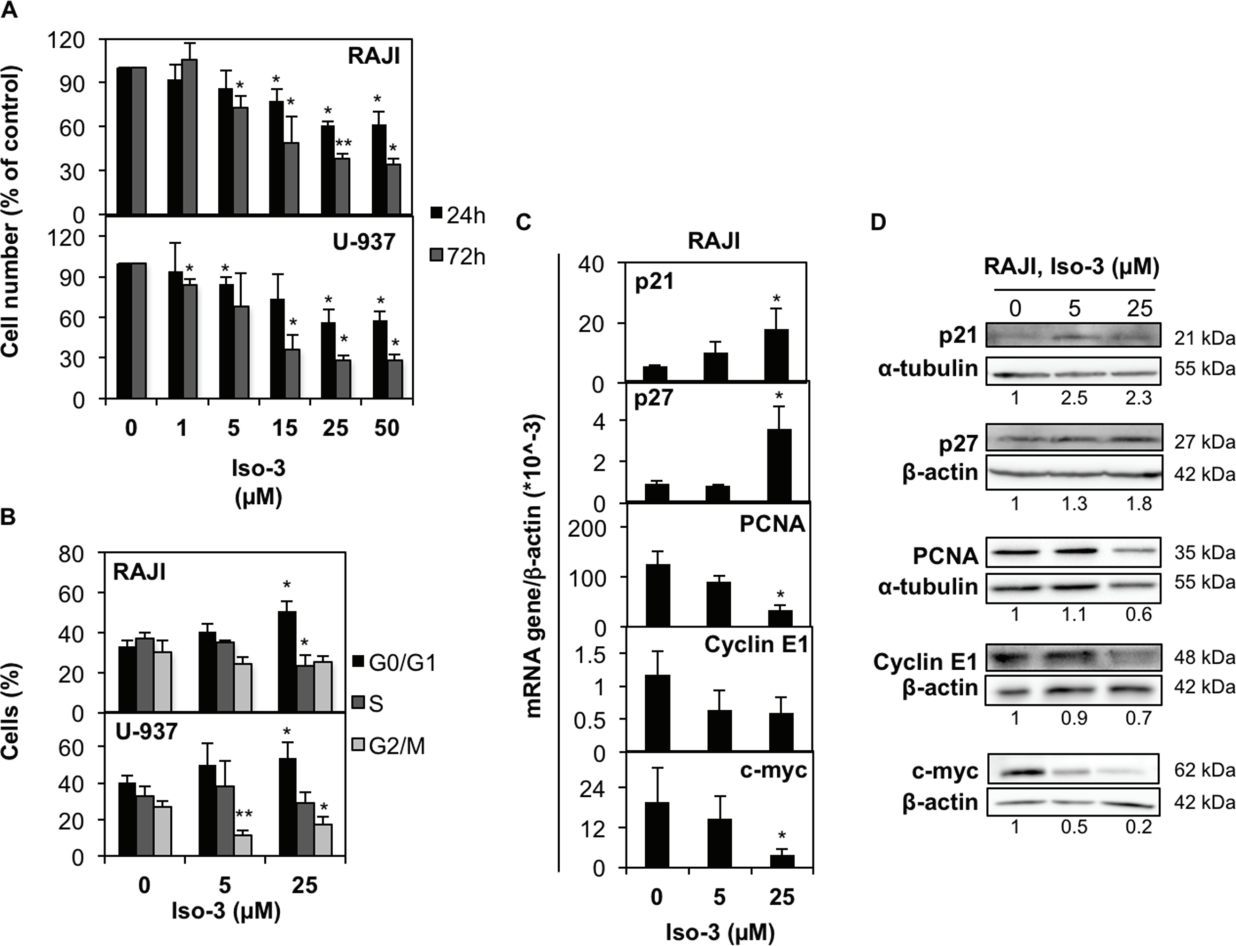
Iso-3 arrests cancer cells in the G0/G1 phase of cell cycle (**A**) RAJI and U-937 cells were treated with Iso-3 and cell number was evaluated after 24 and 72 h. (**B**) RAJI and U-937 cells treated with Iso-3 for 24 h were analyzed for DNA content by flow cytometry. Bars represent the percentage of cells in each cell cycle phase. (**C**) mRNA and (**D**) protein expression levels of cell cycle-related genes in RAJI cells treated with Iso-3 for 24 h. Histograms represent the mean ± SD of three independent experiments. Blots are representative of three independent experiments.

To ascertain the anticancer potential of Iso-3, we treated RAJI and U-937 lymphoma cells with increasing doses of compound. We found a marked reduction of cell proliferation upon Iso-3 treatment in both cell lines (Figure 3A). Proliferation was similarly affected in a broader panel of cancer cell lines (Table 1). By performing cell cycle analysis in RAJI and U-937 cells, we could detect an arrest in the G0/G1 phase of the cell cycle for both cell lines after 24 h of Iso-3 treatment (Figure 3B and Supplementary Figure S4) with an increase of 55 and 32.8% cells in G1 phase at 25 μM, for RAJI and U-937, respectively. Analysis of genes responsible for cell cycle regulation revealed an increase of p21 and p27 mRNA levels in RAJI cells (3.3- and 3.8-fold increase, respectively); concomitantly, Iso-3 produced a decrease of proliferating cell nuclear antigen (PCNA, 3.6 fold), cyclin E1 (1.6 fold) and c-myc (5.1 fold) mRNA expression levels after 24 h of treatment (Figure 3C). Analysis of protein expression levels confirmed these results (Figure 3D). These data are in line with the G0/G1 arrest observed.

**Table 1:** Effect of Iso-3 on the proliferation of various cancer cell lines

Cell line	GI_50_ (μM, 72 h)
RAJI	9.9 ± 8.6
U-937	8.1 ± 5.6
JURKAT	10.2 ± 5.8
K-562	8.3 ± 3.6
MEG-01	14.8 ± 5.3
HL-60	8.1 ± 4.7
SH-SY5Y	> 50^$^
PC-3	8.1 ± 4.4
MDA-MB-231	7.3 ± 7.0

$ 41.1 ± 7.7% growth inhibition at 50 μM.

Data are the mean ± SD of growth inhibitory dose 50% (GI_50_) values calculated from at least three independent experiments.

### Isofistularin-3 induces morphological changes and autophagy in RAJI cells

**Figure 4:**
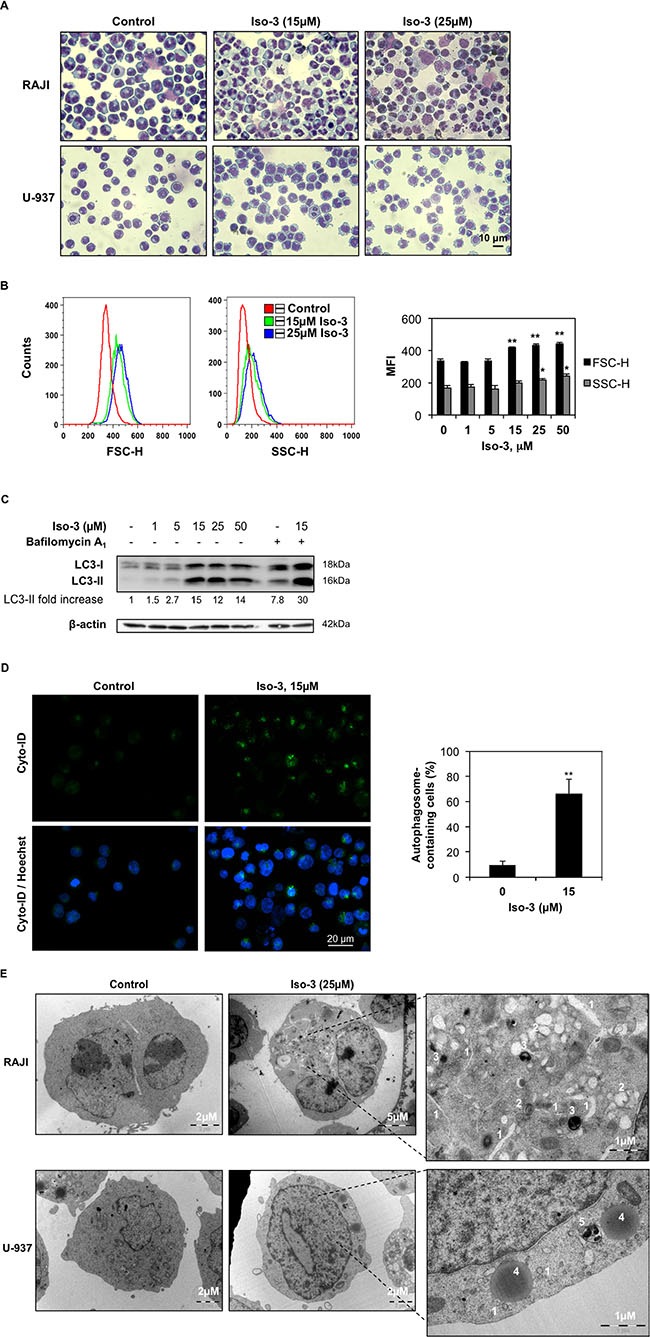
Iso-3 induces autophagy in lymphoma cells (**A**) Morphological analysis was performed by Diff-Quik staining and microscopic observation of RAJI and U-937 cells after 24 h of treatment with Iso-3. (**B**) Cellular size (forward scatter, FSC) and granularity (side scatter, SSC) were measured by flow cytometry in RAJI cells treated for 24 h with Iso-3. (**C**) Western blot analysis of LC3 conversion in RAJI cells treated with different concentrations of Iso-3 for 24 h. Where indicated, bafilomycin A_1_ (40 nM) was added 2 h before harvesting. Blots are representative of three independent experiments. (**D**) RAJI cells where treated or not with Iso-3 for 24 h, then stained with Cyto-ID^®^ Green dye as described in the materials and methods section, and appearance of autophagosome-related vesicles was observed by fluorescence microscopy. Representative images and quantification of autophagosome-positive cells are provided. (**E**) Representative images of electron microscopy analysis of RAJI and U-937 cells treated or not for 24 h with Iso-3. (1) Phagophores, (2) autolysosomes of different maturity, (3) residual bodies, (4) lysosomes, (5) multivesicular body. All histograms represent the mean ± SD of three independent experiments. All blots are representative of three independent experiments.

Furthermore, we performed cellular morphology analysis by microscopic observation in RAJI and U-937 cells. After 24 h of treatment RAJI cells show negligible viability decrease (< 10% trypan blue-positive cells) but overt morphological changes characterized by a strong size increase and the appearance of cytoplasmic vacuoles (Figure 4A). A marked increase in size, and to a lesser extent in granularity, was confirmed by quantification of FSC and SSC parameters by flow cytometry (Figure 4B). Morphological changes were less evident in U-937 cells (Figure 4A). These features prompted us to investigate the appearance of autophagic markers upon Iso-3 treatment. LC3 conversion was assessed by Western blotting and revealed appearance of the converted LC3-II form in RAJI cells exposed to different Iso-3 concentrations for 24 h. Co-treatment of cells with the inhibitor of late steps of autophagy, bafilomycin A_1_, further enhanced the signal (Figure 4C). Moreover, treatment with 15 μM Iso-3 for 12 h revealed accumulation of LC3-II in the presence of bafilomycin A_1_, suggesting an early induction of the autophagic flux (Supplementary Figure S5A). Fluorescence microscopy analysis of RAJI cells stained with Cyto-ID^®^ allowed quantification of vacuoles associated with the autophagic pathway; the percentage of cells presenting autophagic vesicles was 7 times higher in Iso-3-treated cells than in control cells (Figure 4D). To further confirm autophagy induction, we performed transmission electron microscopy analysis of cells after 24 h of treatment with Iso-3. The analysis of cellular structures [39] revealed the appearance of an extensive autophagocytic vacuolization in RAJI cells (Figure 4E), leading in extreme cases to autophagic cell death, characterized by depletion of organelles and absent or non-pyknotic nuclei (Supplementary Figure S5B). The appearance of autophagic features was less pronounced in U-937 cells (Figure 4E).

### Isofistularin-3 induces caspase-dependent and -independent cell death

**Figure 5:**
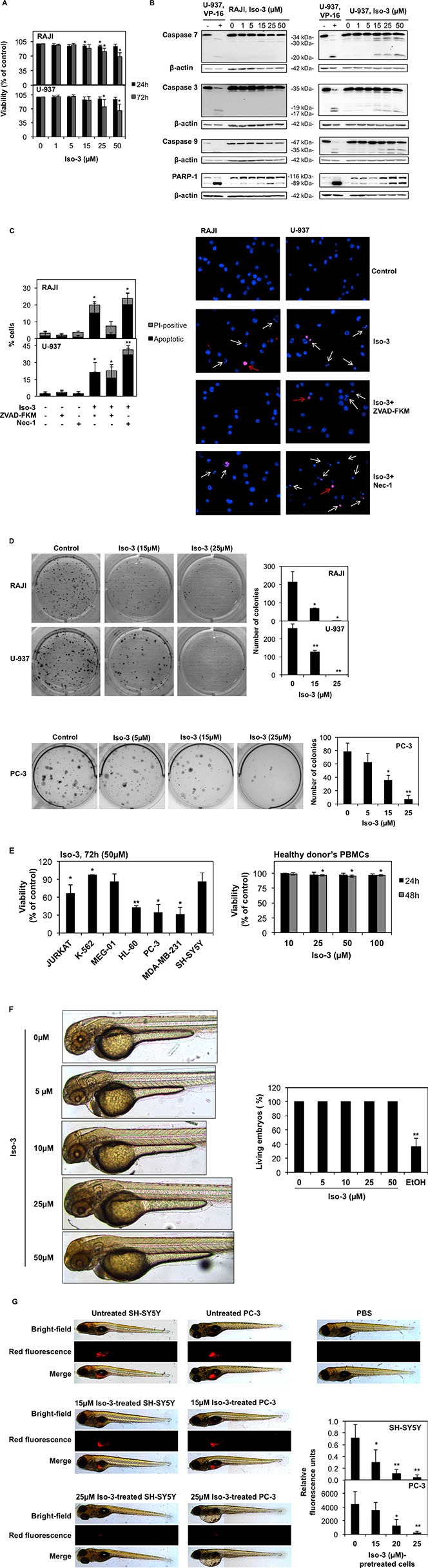
Effect of Iso-3 on cell viability (**A**) The viability of RAJI and U-937 cells was measured by trypan blue exclusion assay after 24 and 72 h of exposure to Iso-3. (**B**) Western blot analyses of caspase activation and PARP-1 cleavage in RAJI and U-937 cells treated for 72 h with Iso-3. U-937 cells, untreated or treated with 100 μM VP-16 for 3 h, were used as negative and positive controls for caspase cleavage, respectively. (**C**) Hoechst-PI staining of RAJI and U-937 cells treated with 50 μM Iso-3 for 72 hours. Apoptotic and non-apoptotic PI-positive nuclei are reported as a percentage of the total number of cells. ZVAD-FKM (50 μM) and Necrostatin-1 (Nec-1; 30 μM) were added 1 h before Iso-3 treatment, where indicated. White arrows: apoptotic cells. Red arrow: PI-positive cells. Pictures are representative of three independent experiments (**D**) RAJI, U-937 and PC-3 cells were grown in the presence of Iso-3 for 10 days and colony formation was then scored. (**E**) Trypan blue scored viability of a panel of cancer cell lines (left) and of PBMCs from healthy donors after Iso-3 treatment at the indicated time points and doses. (**F**) Representative images of Zebrafish embryos after 24 h treatment with the indicated Iso-3 doses (right panel) and corresponding quantification of viable embryos percentage (left panel). Ethanol 3% (EtOH) was used as a positive control for toxicity. (**G**) Fluorescent SH-SY5Y or PC-3 cells were treated or not *in vitro* at different concentrations of Iso-3 for 24 h and then injected in the zebrafish yolk sac. Fluorescence was quantified. Representative images from a total of six to nine fish per condition. Fluorescence intensity quantification graphs are shown. PBS injection was used as a control for injection toxicity. All histograms represent the mean ± SD of three independent experiments. All blots are representative of three independent experiments.

It is well known that prolonged proliferation arrest and/or onset of autophagy may trigger cell death. Thus, we tested the effect of Iso-3 on cell viability after extended periods of treatment. Trypan blue analysis of RAJI and U-937 cells revealed a fraction of cells starting to undergo cell death after 72 h of treatment (Figure 5A). Western blot analysis for caspase and poly-(ADP-ribose) polymerase-1 (PARP-1) cleavage showed robust cleavage in U-937 cells at the highest Iso-3 concentration, whereas a weaker cleavage was observed in RAJI cells (Figure 5B). Subsequently, we investigated RAJI and U-937 nuclear morphology by Hoechst-propidium iodide (PI) staining after 72 h of treatment with 50 μM Iso-3, in presence of the pan-caspase inhibitor ZVAD-FKM or the necroptosis inhibitor Necrostatin-1, respectively. Results show the appearance of apoptotic nuclei in both cell lines, and of a fraction of non apoptotic-PI positive nuclei in RAJI cells. The pan-caspase inhibitor ZVAD-FKM was able to prevent apoptotic cell death in RAJI but not in U-937 cells, suggesting a switch to a caspase-independent type of apoptosis upon prolonged Iso-3 treatment in this cell line. Necrostatin-1 failed to prevent Iso-3-induced cell death in any of the cell lines tested (Figure 5C). To confirm the potential of Iso-3 to impair the replicative ability of cancer cells, we investigated RAJI, U-937 and prostate cancer PC-3 cells colony formation ability in the presence of increasing concentrations of compound. Colony formation was strongly reduced by Iso-3 at 15 μM and almost completely abolished at 25 μM, in all cell lines (Figure 5D).

Viability was also decreased to various extents in a panel of other cancer cell lines, but not in peripheral blood mononuclear cells (PBMCs) isolated from healthy donors (Figure 5E). Furthermore, we provide evidence that Iso-3 did not trigger acute toxicity in the context of zebrafish development, as shown by the absence of morphological defects and no occurrence of dead embryos, even at the highest concentration used, after 24 h of treatment (Figure 5F).

To extend our colony formation assays to an *in vivo* setting, we compared the capacity of Iso-3 to abrogate tumor formation in a zebrafish xenograft model. Our results show a dose-dependent inhibition of tumor formation when we injected Iso-3-pretreated fluorescent PC-3 and SH-SY-5Y cancer cells (Figure 5G and Supplementary Figure S6) thus validating our *in vitro* results.

### Isofistularin-3 sensitizes lymphoma cells to TRAIL-induced apoptosis

**Figure 6:**
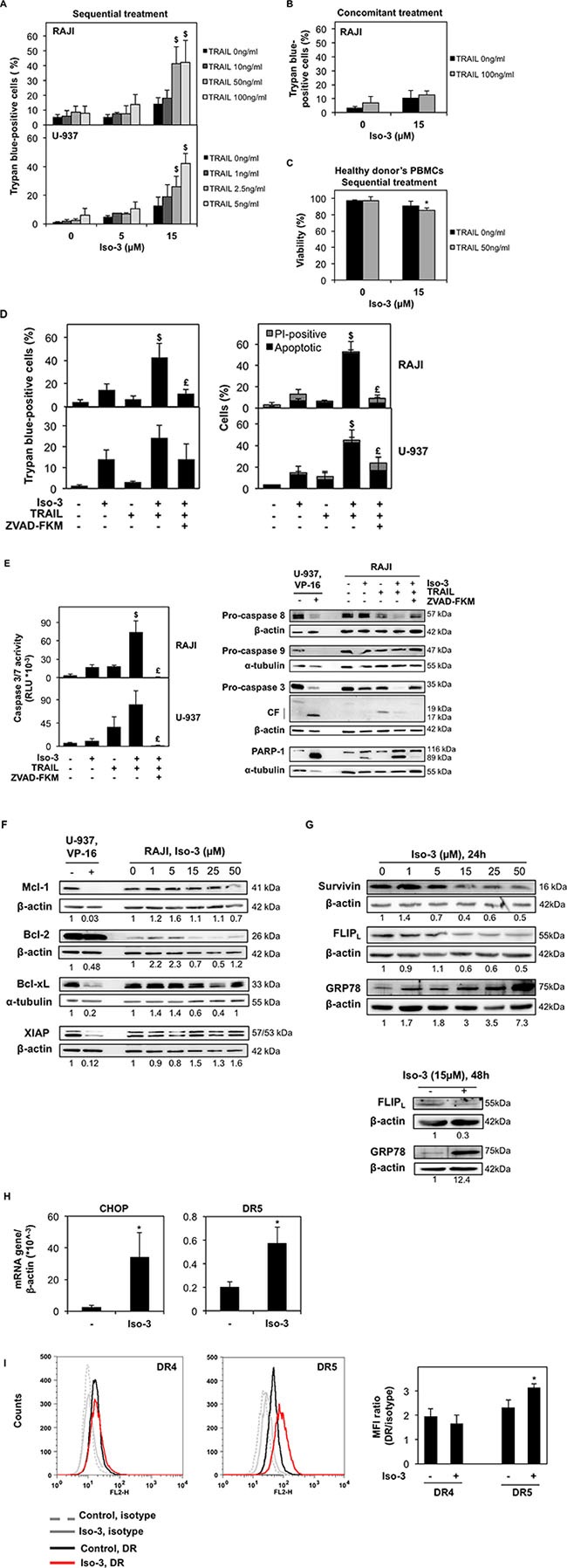
Iso-3 sensitizes cancer cells to TRAIL-induced apoptosis (**A**) RAJI and U-937 cells were treated with the indicated concentrations of Iso-3 during 24 h, then increasing concentrations of TRAIL were added for additional 24 h and cell viability was assessed. Significant differences between combination treatments, untreated controls and single agents are indicated with a $. (**B**) Viability of RAJI cells co-treated with the indicated doses of Iso-3 and TRAIL for 48 h. (**C**) Healthy donors' PBMCs were treated with Iso-3 during 24 h, then TRAIL was added for additional 24 h and viability was measured. (**D**) RAJI and U-937 cells were treated with Iso-3 (15 μM) and Z-VAD-FKM (50 μM) for 24 h, before TRAIL addition for 24 h (50 ng/ml for RAJI, 5 ng/ml for U-937). Cell death was measured by trypan blue exclusion assay (left panel) and Hoechst-PI staining (right panel). Significant differences between combination treatments, untreated controls and single agents are indicated with a $; significant differences between combination treatments with and without ZVAD-FKM are indicated with a £. (**E**) Cells were treated as in panel D and caspase activation was measured by luminescent caspase 3/7 assay (right panel). Significant differences are indicated as in panel D. Western blot analysis of caspases and PARP-1 cleavage was performed in RAJI cells (left panel). CF = cleaved fragments. (**F**) Expression levels of a panel of anti-apoptotic proteins implicated in TRAIL resistance in RAJI cells after 24 h treatment with Iso-3. (**G**) Survivin, FLIP and GRP78 expression levels after 24 h of Iso-3 treatment (upper panel); FLIP and GRP78 levels after 48 h of 15 μM Iso-3 treatment (lower panel) in RAJI cells. Blots are representative of three independent experiments. (**H**) mRNA expression levels of CHOP and DR5 in RAJI cells after treatment with 15 μM Iso-3 for 48 h. (**I**) FACS analysis of DR4 and DR5 surface levels in RAJI cells after 48 h treatment with 15 μM Iso-3. Representative histograms (left panel) and quantification of mean fluorescence intensity (MFI) levels (right panel) relative to matched control PE-conjugated IgG antibodies. All histograms represent the mean ± SD of three independent experiments. All blots are representative of three independent experiments.

Combination treatments represent a promising and increasingly pursued strategy in anticancer therapy, especially in cancer presenting TRAIL resistance. As G0/G1 cell cycle arrest was previously reported to sensitize cancer cells against TRAIL [40] and epigenetic modulators were often reported to be TRAIL sensitizers [41, 42], we assessed the effect of Iso-3 on cancer cell viability after combination treatments with TRAIL. We pre-treated RAJI and U-937 cell lines with sub-toxic concentrations of Iso-3 for 24 hours, then we treated cells at indicated doses of TRAIL and measured the effects 24 hours after addition. TRAIL concentrations were chosen according to the differential sensitivity of the two cell lines reported in the literature and verified by own work. Results revealed that the viability was not significantly affected by TRAIL or Iso-3 treatments alone (Figure 6A). Conversely, pre-treatment with Iso-3 followed by TRAIL exposure produced a marked decrease of cell viability for both models (Figure 6A). Combination index (CI) [43] shows synergism for these two agents at several dose combinations (Table 2). The Iso-3 dose of 15 μM resulted in the strongest synergism (CI = 0.22 with 50 ng/ml TRAIL in RAJI; CI = 0.21 with 5 ng/ml TRAIL in U-937). In contrast, simultaneous treatment of RAJI cells with both drugs failed to affect cell viability, suggesting that early effects produced by Iso-3 are necessary for TRAIL sensitization (Figure 6B). Importantly, we showed that Iso-3 pre-treatment did not sensitize PBMCs from healthy donors to TRAIL (Figure 6C). Subsequently, we analyzed the typology of cell death produced by the combination treatment, focusing on the most interesting combination doses (Figure 6D). The results of nuclear morphology analysis revealed the induction of apoptosis in both cell lines. Activation of effector caspases (3/7) was confirmed by caspase activity assay (Figure 6E, right panel) and western blot analysis revealed that caspase 8, caspase 3 and PARP-1 were efficiently cleaved after Iso-3 plus TRAIL treatment in RAJI cells, as shown by a strong decrease of pro-caspase bands and appearance of active caspase 3 fragments. Conversely, no decrease of the pro-caspase 9 was recorded, suggesting that the extrinsic apoptotic pathway alone was implicated in the induced cell death (Figure 6E, left panel). Apoptosis induction was further confirmed by the concomitant use of the pan-caspase inhibitor ZVAD-FKM, which greatly reduced the amount of cell death and prevented caspase activation, as well as PARP cleavage (Figure 6D, 6E, 6F).

**Table 2:** Combination index (CI) for Iso-3 plus TRAIL treatments, in U-937 and RAJI cells

Cell Line	Concentration Iso-3 (μM)	Concentration TRAIL (ng/ml)	CI
U-937	5	1	0.77
	15	2.5	0.43
	15	5	0.21
RAJI	5	100	0.36
	15	50	0.22
	15	100	0.21

Cells were pre-treated with Iso-3 for 24 h before addition of TRAIL for an additional 24 h. CI is calculated from mean values of at least three independent trypan blue exclusion experiments.

To investigate the mechanism of TRAIL sensitization by Iso-3, we first analyzed expression levels of several proteins known to play important roles in TRAIL resistance [44]. First, we did not detect any changes in the expression levels of anti-apoptotic Bcl-2 family proteins, nor of the inhibitor of apoptosis (IAP) X-linked-IAP (XIAP) (Figure 6F). In contrast, we detected a decrease in the expression levels of the IAP survivin as well as of cellular FLICE-like inhibitory protein (FLIP) (Figure 6G). FLIP down-regulation was previously reported under endoplasmic reticulum (ER) stress conditions [45, 46] and many ER stress-inducing drugs were shown to sensitize cancer cells to TRAIL [47]. Thus, we verified levels of the ER chaperone GRP78 upon Iso-3 treatment, as a marker of ER-stress induction. We found a strong dose-dependent increase in GRP78 expression levels (Figure 6G) supporting the idea that Iso-3-treated RAJI cells undergo ER stress. In accordance, treatment of cells with 15 μM Iso-3 for 48 h resulted in increased CCAAT-enhancer-binding *protein* homologous *protein* (CHOP) and death receptor (DR)5 mRNA expression (Figure 6H). Finally, we verified surface expression levels of both functional TRAIL receptors, DR4 and 5. Our results show that treatment with 15 μM Iso-3 increased DR5 surface expression levels by around 30%, whereas DR4 surface levels were unchanged (Figure 6I).

## DISCUSSION

DNMT inhibitors reported in the literature include a variety of molecules with very different chemical structures. Compounds shown to behave as DNMT inhibitors include an increasing number of natural molecules [6–8], all of which raised a huge interest for their ability to demethylate and re-activate specific TSGs in different cell lines. Such effects are believed to participate in the antitumor or tumor-preventive effects of these compounds. We showed here that Iso-3, a brominated alkaloid from the sponge *Aplysina aerophoba*, is able to inhibit DNMT1 activity *in vitro*. Docking studies suggest that Iso-3 is a direct, DNA-competitive, DNMT1 inhibitor. A similar mode of action was recently demonstrated for another natural DNMT inhibitor, laccaic acid A [48]. Despite some structure similarities with the dual HDAC and methyltransferase inhibitor bromotyrosine derivative PsA, we did not find evidence that Iso-3 functions as an HDAC inhibitor. This is most likely due to the absence of the thiol linker moiety present in PsA.

DNMT inhibition should lead to a decrease of DNA methylation at some genetic locus. Here, we show that Iso-3 induces AHR promoter demethylation and restores mRNA re-expression in RAJI cells. AHR is a ligand-activated transcription factor, known for its role in the detoxification from environmental carcinogens. More importantly, AHR is implicated in numerous other cellular functions such as cell cycle regulation and immune functions [27, 49, 50]. Several reports established a tumor suppressive activity of this gene [25–27]. The finding that AHR is hypermethylated and repressed in several acute lymphoblastic leukemia (ALL) cell lines and in patients [28] reinforced the idea that AHR repression contributes to malignancy in these models. Previous work showed that RAJI cells do not express AHR [51]. Here, we report for the first time promoter hypermethylation as a mechanism for AHR repression in a Burkitt's lymphoma model. We also detected for the first time in RAJI cells the presence of a GGGGC(2×) repeat insertion in the Sp1 region of AHR promoter (SNP rs71010234) (Figure 2A), a SNP previously reported for other cancer cell lines and human samples [52–54].

In this study, treatments with Iso-3 led to decrease methylation at specific CG sites in the AHR promoter. Intriguingly, the most affected CG site by Iso-3 is located near Sp1 consensus elements, confirming that methylation of CG sites flanking the Sp1 consensus seem to be most important in affecting Sp1 binding. Our results suggest that Iso-3-induced demethylation contributes to the increase of AHR expression achieved. AHR protein was undetectable in Iso-3- as well as DAC-treated samples despite increased mRNA levels: we hypothesize that in addition to promoter methylation, a translational inhibitory mechanism regulates AHR expression in these cells. Recent work showed that hepatic AHR expression can be regulated by RNA editing mechanisms, creating miRNAs recognition sites in AHR untranslated region (UTR) [55]. We also found that AHR mRNA levels were increased by DAC and Iso-3 in neuroblastoma SH-SY5Y cells, in which AHR is known to be absent [32] by so far non-described mechanisms. These data suggest methylation-mediated AHR repression in SH-SY5Y, similarly to RAJI cells; however, further studies on promoter methylation state in these cells are needed to validate this hypothesis. Moreover, at this point the ability of Iso-3 to affect DNA methylation at the genomic level and in other cell models remains to be investigated.

We also observed that Iso-3 reduced DNMT1 protein levels in RAJI cells, but not in other tested cell lines, whereas DAC depleted the protein in all models. These results are in agreement with a non-covalent interaction of Iso-3 with DNMT1 and suggest a post-transcriptional event leading to protein destabilization, which may occur downstream to its interaction with Iso-3, in RAJI cells. The mechanism underlying this cell line-specific degradation is still unclear; however, DNMT1 stability is regulated by the concerted action of several post-translational modifiers and depends on cell cycle progression [56]. Various levels of expression or activity of DNMT1 partners and regulators in the cell lines tested may be responsible for differential outcomes. Moreover, RAJI are latently infected with Epstein Barr virus (EBV), which was shown to modulate DNMT1 levels [57] and could play a role in Iso-3-induced DNMT1 downregulation. Altogether, we conclude here that alterations in DNMT1 expression levels are not an essential feature of Iso-3 activity, and are not required to achieve the anti-proliferative effect of the drug. Overall, the exact contribution of Iso-3 DNMT1 inhibitory activity in the anticancer effect of the drug remains to be established, and our work does not exclude that other relevant cellular targets exists and contribute to the described biological effects.

So far Iso-3 was poorly characterized with respect to its anticancer activities. In this study we showed that this marine compound displays a broad-range of antiproliferative activity against cancer cells and does not affect viability of healthy donors' PBMCs. A major tumor driver in Burkitt's lymphoma, c-myc [58], is depleted and levels of the c-myc regulated genes p21 and 27 are increased concomitantly with cell cycle arrest in RAJI cells treated by Iso-3. The cytostatic effect of Iso-3 occurs in the first place, with cell death induction appearing only at later time points and at the highest concentrations tested. In line with these features, the potential of Iso-3 to impair the replicative ability of cancer cells has been confirmed by colony formation assay and by zebrafish injection of *in vitro* Iso-3 treated cancer cells.

In an acute toxicity assay, Iso-3 did not reveal toxic effects against zebrafish development. The zebrafish development model has been also used to test the impact of chemicals on DNA methylation. *De novo* methylation of the *no tail* gene leads to abnormal tail development [59] and this feature is altered in response to DAC and other chemicals [60]. We did not found obvious phenotypical changes in zebrafish that could be related to Iso-3 demethylating activity after 24 h of treatment. Since the dynamic of *de novo* methylation seems to be mostly involved in development, our data may suggest that Iso-3 lacks effect on *de novo* methyltransferases DNMT3A ad 3B. However, to exclude any effect on zebrafish development further analyses would be required, looking at specific phenotypical changes that may also occur in later stages of development, such as late hematopoiesis or specific organ developmental impairment [61, 62].

Cytostatic compounds counteract aberrant mechanisms sustaining rapid proliferation, which are mostly specific of cancer cells; thus, this class of molecules could represent a less toxic alternative to purely cytotoxic compounds. In this context, Iso-3 is a new potential candidate for non-toxic anticancer therapy. Moreover, combination therapies may take advantage of cytostatic molecules to lower the dose of cytotoxic drugs to be applied to cancer patients.

Accumulating evidence show that autophagy participates in the anticancer effect of epigenetic modulators. We previously showed that the DNA demethylating agent DAC induces autophagy in chronic myeloid leukemia [19]; recently, autophagy was implicated in the anticancer effect of zebularine, another DNA demethylating agent, against colorectal cancer cells [63]. Moreover, HDAC inhibitor treatment was shown to elicit autophagic cell death in several cancer cell models [64] and the histone methyltransferase inhibitor BIX-01294 recently showed the same ability in breast cancer cells [65].

Interestingly, epigenetic drugs have been already shown to synergize with chemotherapy and some are now undergoing several clinical trials in combination treatments. In particular, both demethylating agents and HDAC inhibitors demonstrated the ability to overcome TRAIL resistance through various mechanisms [41, 42]. Importantly, pre-treatment with Iso-3 resulted in a strong sensitization of resistant cells to apoptosis induced by TRAIL. Therapeutic antibodies targeting TRAIL receptors are undergoing clinical trials and represent a very promising strategy to kill cancer cells without harming normal cells: however many cancers are resistant to these agents, and strong efforts are undergoing to find non-toxic molecules sensitizing cancer cells to TRAIL. Cell cycle arrest was previously reported to affect sensitivity of cancer cells to TRAIL [40, 66]; interestingly, we show here that only pre-treatment with Iso-3, and not concomitant treatment with Iso-3 and TRAIL, sensitizes RAJI cells to the latter, supporting a role for G0/G1 arrest in sensitization. Healthy donors' PBMCs were not affected by the combination treatments, suggesting that Iso-3 could provide a non-toxic strategy to overcome TRAIL resistance. Several Burkitt's lymphoma cell lines have been previously shown to display TRAIL resistance, by a mechanism that was reported to involve impaired activation of caspase 8 [67]. In accordance, we show here that the sensitization induced by Iso-3 in RAJI cells involves re-activation of caspase 8 but not caspase 9. Moreover, anti-apoptotic Bcl-2 family proteins and XIAP levels were not affected by Iso-3, whereas survivin levels were depleted after 24 h of treatment. Interestingly, besides its caspase inhibitory activity, survivin plays an important role in cell cycle progression [68]. In addition, sub-toxic concentrations of Iso-3 effectively decreased protein levels of cFLIP, a well-recognized inhibitor of caspase-8 activation, which is frequently overexpressed in TRAIL-resistant cancer cells [69]. Concomitantly, we revealed a strong up-regulation of the ER stress induction marker GRP78. ER stress was frequently shown to restore TRAIL sensitivity by promoting, among other responses, cFLIP down-regulation [45, 46] and TRAIL receptor DR5 up-regulation. [47, 70–72]. In agreement with an ER stress-mediated effect, our results show that DR5 but not DR4 surface levels are increased after Iso-3 treatment. Finally, autophagy, which could result as a consequence of sustained ER stress, may also participate in the observed TRAIL sensitization, by providing a platform for DISC formation and caspase 8 activation [73]. Altogether, these data support a restoration of the extrinsic apoptotic pathway responsiveness by Iso-3, with minor or no involvement of the mitochondrial pathway, and with an ER-stress signature. The exact mechanism and the relative contribution of each player in the Iso-3-induced cancer cell sensitization to TRAIL remains to be elucidated, as well as the correlation with Iso-3 epigenetic activity, for which further investigation is guaranteed.

In conclusion, Iso-3 is a new promising agent impacting on cancer epigenetics, with a strong antiproliferative activity against cancer cell lines, and the potential to sensitize cancer cells to molecules triggering the extrinsic apoptotic pathway.

## MATERIALS AND METHODS

### *In vitro DNMT* and *HDAC* activity assays

DNMT1 activity was measured using the *in vitro* DNMT activity/inhibition assay (Active Motif, Rixensart, Belgium) according to manufacturer's instructions. The methylation reaction was performed by incubating 5 ng of purified DNMT1 for each condition with increasing concentrations of compounds for 2 hours. The methylated DNA was then recognized by the His-tagged methyl-CpG binding domain protein 2b. The addition of a poly-histidine antibody conjugated to horseradish peroxidase then provided a colorimetric readout quantified with a spectrophotometer (SpectraCount, Packard) at the wavelength of 450 nm. TritonX-100 (0.01%) was added to the assay from a fresh dilution where indicated. For total DNMT activity assay, RAJI nuclear extracts were used as DNMT source. Total *in vitro* HDAC activity was measured as previously described [14].

### Docking studies

Initial structures of DNMT1 were obtained from the Protein Data Bank (PDB; PDB ID: 3SWR) and coordinates for the sinefungin, Iso-3, aerothionin, and bisoxasolidinone were generated using the GlycoBioChem PRODRG2 Server [74]. After removing ligands and DNA in the original data, we operated the PatchDock program [22, 74] with protein and compound as receptor and ligand, respectively. Structural superposition of 3PTA and 3SWR coordinates was performed using WinCoot [66].

### Cell culture and reagents

RAJI (Burkitt's lymphoma), U-937 (non-Hodgkin lymphoma), JURKAT (ALL), K-562 (chronic myeloid leukemia, CML), HL-60 (promyelocytic leukemia) MEG-01 (CML in megakaryocytic blast crisis), PC-3 (prostate cancer) cells were obtained from the Deutsche Sammlung von Mikroorganismen und Zellkulturen GmbH (Braunschweig, Germany). SH-SY5Y (neuroblastoma) and MDA-MB-231 (breast adenocarcinoma) were obtained from the American tissue culture collection (Manassas, VA, USA). All cell lines were cultured in RPMI 1640 medium (Lonza, Verviers, Belgium) supplemented with 10% heat-inactivated fetal calf serum (Lonza) and 1% antibiotic–antimycotic (Lonza). Peripheral blood mononuclear cells (PBMCs) from human healthy donors were isolated and cultured as previously described [19]. All experiments were performed on cells in the exponential growth phase. Iso-3 was purified from *Aplysina aerophoba* [17] and dissolved in DMSO. Epigallocatechin gallate (EGCG), DAC, Necrostatin-1, VP-16, PP242 and bafilomycin A_1_ were purchased from Sigma-Aldrich (Bornem, Belgium). SAHA and Z-VAD-FKM were purchased from Cayman Bio-connect (Huissen, The Netherlands) and from Millipore (Merck, Brussels, Belgium), respectively. All drugs were dissolved in DMSO. Recombinant human TRAIL was purchased by Enzo Life Science (Antwerpen, Belgium).

### mRNA expression analysis

Gene expression analyses were performed by real-time RT-PCR as previously described [75]. Primer sequences are available on request.

### CpG methylation analysis

Bisulfite cloning and sequencing was performed as previously described [76]. Briefly, BSP primers specific for AHR (NCBI accession number NC_000007.14) were designed with Methylprimer express software (Applied Biosystems, Halle, Belgium). CpG island search was performed with the following parameters: a minimum length of 300 nucleotide, minimum G+C content of 50%, observed/expected CpG ratio of 0.6. A 1489-nt fragment was identified, containing a CpG island and the transcription start site. Within this fragment, a 320-nucleotide region was selected for further analysis. Bisulfite-modified genomic DNA was amplified with specific primers (FW: 5′-GGGTGGGGTTTTTAAGGA-3**′**; REV: 5′-CTTCCTAAATCCAAAATACTTCC-3**′**) and the Hot start TaqTM polymerase (Qiagen, Venlo, The Netherlands). Thermal conditions were 15 min at 95¼C, 40 cycles of 94¼C for 1 min, 58¼C for 1 min, 72¼C for 1 min, and a final extension step of 10 min at 72¼C. PCR products were separated on agarose gels, purified with the Qiaquick Gel extraction Kit (Qiagen), cloned into pCR2.1-TOPO vector (Invitrogen, Tournai, Belgium). Plasmids were purified with Qiaprep Spin miniprep kit (Qiagen) and sequenced (GATC Biotech, Konstanz, Germany). Methylation analysis was performed by QUMA software [77].

### Cell proliferation and viability assays

Cell proliferation and viability were measured by using the trypan blue exclusion assay (Lonza, Verviers, Belgium). The number of cells per ml was counted and the fraction of trypan blue-positive cells was estimated by using a Cedex cell counter (Innovatis AG, Roche, Luxembourg, Luxembourg). For colony formation assays, cells (10^3^ cells/ml) were grown in semi solid methylcellulose medium (Methocult H4230, StemCell Technologies Inc., Vancouver, Canada) supplemented with the indicated Iso-3 concentrations. Colonies were detected after 10 days of culture by adding 1 mg/ml of 3-(4,5-dimethylthiazol-2-yl)-2,5-diphenyltetrazoliumbromide (MTT) reagent (Sigma) and were scored by Image J software (U.S. National Institute of Health, Bethesda, MD, USA).

### Evaluation of apoptosis

The percentage of apoptotic cells was quantified as the fraction of cells showing apoptotic, fragmented nuclei, as assessed by fluorescence microscopy (Leica-DM IRB microscope, Lecuit, Howald, Luxembourg) after staining with Hoechst 33342 and PI. Enzymatic activity of caspases-3/7 was determined using the Caspase-Glo 3/7 Assay (Promega, Leiden, The Netherlands). The assay was performed according to the manufacturer‘s instructions and luminescence was measured using an Orion Microplate Luminometer (Berthold, Pforzheim, Germany).

### Cell cycle distribution and size/granularity analyses

For cell cycle analysis, cells were collected and fixed in ethanol 70%. DNA was stained with a PI solution (1 μg/ml, Sigma-Aldrich) in 1x PBS, supplemented with RNase A (100 μg/ml; Roche). Samples were analyzed by flow cytometry (FACS Calibur, Becton Dickinson (BD) Biosciences, San Jose, CA, USA). Relative cell size and granularity were evaluated based on forward (FSC) and side (SSC) scatter parameters assessed by flow cytometry. Data were recorded statistically (10,000 events/sample) using the CellQuest software (BD Biosciences) and analyzed using Flow-Jo 8.8.5 software (Tree Star, Inc., Ashland, OR, USA).

### Protein extraction and western blotting

Whole cell extracts were prepared using M-PER^®^ (Thermofisher, Erembodegen, Belgium) supplemented by 1x protease inhibitor cocktail (Complete EDTA-free, Roche, Prophac, Luxembourg, Luxembourg) according to manufacturer's instructions. Histone enrichment was performed as previously described [14]. Western blots were performed using the following primary antibodies: anti-DNMT3A (3598), anti-caspase 7 (9494S), anti-caspase 9 (9502S), anti-caspase 8 (9746), anti-PARP (9542), anti-Mcl-1 (4572S), anti-cyclin E1 (4129) from Cell Signaling (Leiden, The Netherlands); anti-DNMT1 (sc-10222), anti-caspase 3 (sc-56053), anti-p21 (sc-817), anti-p27 (sc-527), anti-PCNA (sc-9857R), anti-GRP78 (13968), anti-AHR (8088) from Santa Cruz Biotechnology (Boechout, Belgium); anti-c-myc (51–1485GR), anti-Bcl-xL (610212), anti-XIAP (610763) from BD Pharmigen (Erembodegem, Belgium); anti-FLIP (804–961) from Enzo Life Science, anti-DNMT3B (2851) from Abcam (Cambridge, UK); anti-acetylated histone H4 (06–866), anti-histone H1 (05–457), anti Bcl-2 (OP60) and anti-alpha tubulin (CP06) from Millipore; anti LC-3 (L7543) and anti-beta actin (5441) from Sigma Aldrich; anti-survivin (AF886) from R & D System (Abingdon, UK). Bands were quantified using ImageQuant TL (GE Healthcare, Buckinghamshire, UK) and values of fold change are reported underneath western blots.

### Morphological analysis

Cells (3 × 10^5^) were washed in 1x PBS and spun onto a glass slide using a Shandon Cytospin 4 (Thermofisher), fixed and stained with the Diff-Quick stain kit (Dade Behring S.A., Brussels, Belgium) according to the manufacturer's procedure. Images were acquired using a Leica DM2000 equipped with a DFC420C camera and Leica FireCam software.

### Analyses of autophagic vesicles

Samples for transmission electron microscopy analysis were prepared and observed as previously described [19]. Current guidelines for the analysis of cellular structures were followed for interpretation [39]. For fluorescence microscopy analysis, 1 × 10^6^ cells were stained with Cyto-ID^®^ Green dye and Hoechst 33342, according to manufacturer's instructions (Enzo Life Science). Cells were observed by fluorescence microscopy analysis using an IX81(MT10) Olympus microscope (Olympus, Aartselaar, Belgium).

### Zebrafish toxicity assay and cancer cell xenografts

Wild type zebrafish (*Danio rerio*) were obtained from the Zebrafish International Resource Center (ZIRC, University of Oregon, OR), maintained according SNU guidelines at 28.5°C with 10 hr dark/14 hr light cycles. For toxicity assays, embryos were treated with 0.003% phenylthiourea 14 hr before the assay in order to remove pigmentation. Two hr before the assay, the embryo's shell was eliminated and then treated for up to 24 hr with Iso-3 at indicated concentrations in 24 well plates. Ethanol (3%) was used as a positive control for toxicity. Viability and abnormal development were assessed after 24 hr of treatment under light microscopy (Carl Zeiss Stereo microscope DV4, Seoul, Korea). Pictures were taken by fixing zebrafish embryos onto a glass slide with 3% methyl-cellulose (Sigma Aldrich). For cancer xenograft assays, after mating, fertilized eggs were incubated in Danieau's solution with 0.003% of phenylthiourea (PTU) at 28.5°C for 48 hr. Micropipettes for injection and anesthesia were generated from a 1.0 mm glass capillary (World Precision Instruments, FL, USA) by using a micropipette puller (Shutter Instrument, USA). 48 hours post fertilization (hpf), zebrafish were anesthetized in 0.02% tricaine (Sigma, MO) and immobilized on an agar plate. 100–200 of Vampiro-PC3 or Vampiro-SH-SY5Y cells (Innoprot, Spain), with and without Iso-3 pretreatment at indicated concentrations for 24 hr, were injected into the yolk sac by microinjection (PV820 microinjector, World Precision Instruments, FL, USA). Subsequently, zebrafish were incubated in 96-well plates containing Danieau's solution with 0.003% phenylthiourea (PTU) at 28.5°C for 72 hr. Fishes were then immobilized in a drop of 3% methylcellulose in Danieau's solution on a glass slide. Pictures were taken by fluorescence microscopy (Leica DE/DM 5000B). Area of fluorescent tumors was quantified by Image J software (http://rsb.info.nih.gov/ij/docs/index.html).

### Analysis of cell surface expression of DR4 and DR5

Control and treated RAJI cells (1 × 10^6^) were stained with phycoerythrin (PE)-conjugated mouse monoclonal anti-human DR4 and DR5 antibodies (R & D system) or matched PE-conjugated mouse IgG, for 1 h at 4¼C. Cells were then resuspended in 1 × PBS and analyzed by flow cytometry. Data were analyzed by using Flow-Jo 8.8.5 software (Treestar, Ashland, OR, USA).

### Statistics

Significant differences were determined using the Student's *t*-test or the Fisher exact test (for methylation data). Statistical significances were evaluated at *p*-values below 0.05 and represented by the following legend: *p ≤ 0.05, **p ≤ 0.001. Growth inhibitory dose 50% (GI_50_) calculation was performed with Prism software. Combination index (CI) was calculated according to Chou and Talalay [43] using Compusyn Software (ComboSyn, Inc., Paramus, NJ, USA). CI values below 1 indicate synergism. All histograms represent the mean ± SD of at least 3 independent experiments.

## SUPPLEMENTARY MATERIALS FIGURES AND TABLE


