# Supplementary material for: Discovery and characterization of Isofistularin-3, a marine brominated alkaloid, as a new DNA demethylating agent inducing cell cycle arrest and sensitization to TRAIL in cancer cells
Source: Oncotarget. 2016 Mar 19;7(17):24027–49. doi: 10.18632/oncotarget.8210 (PMC5029682; doi:10.18632/oncotarget.8210)
Supplement: Supplementary file 1 [file oncotarget-07-24027-s001.pdf]

Discovery and characterization of Isofistularin-3, a marine brominated alkaloid, as a new DNA demethylating agent inducing cell cycle arrest and sensitization to TRAIL in cancer cells

Supplementary Materials

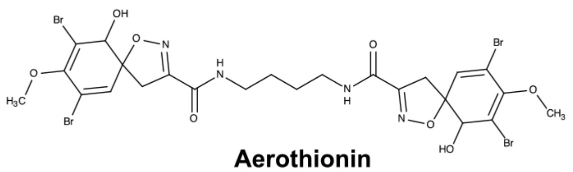

Aerothionin

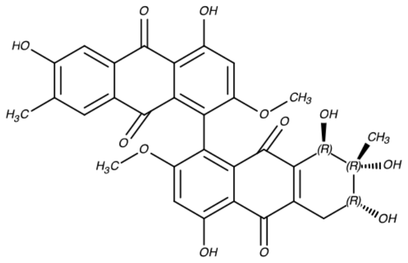

Alterporriol F

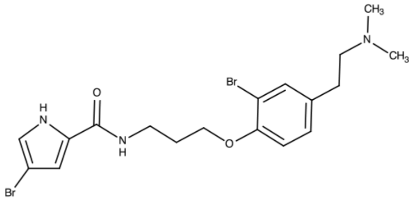

Agelanesin A

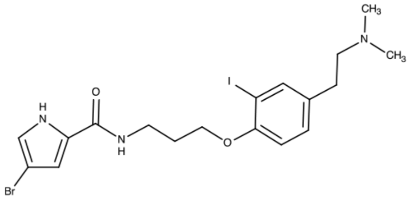

Agelanesin B

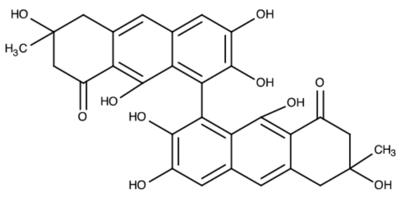

Talaromanin B

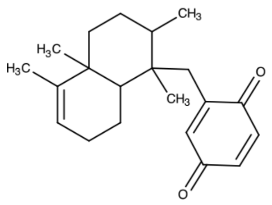

Avarone

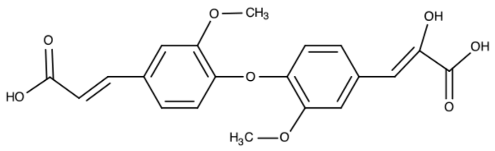

AM7b 5.3.7.6

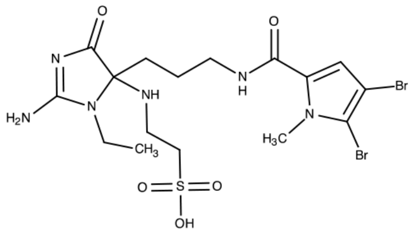

Mauritamide C

Supplementary Figure S1: Chemical structure representation of hit compounds found to inhibit *in vitro* DNMT1 activity in Supplementary Table S1.

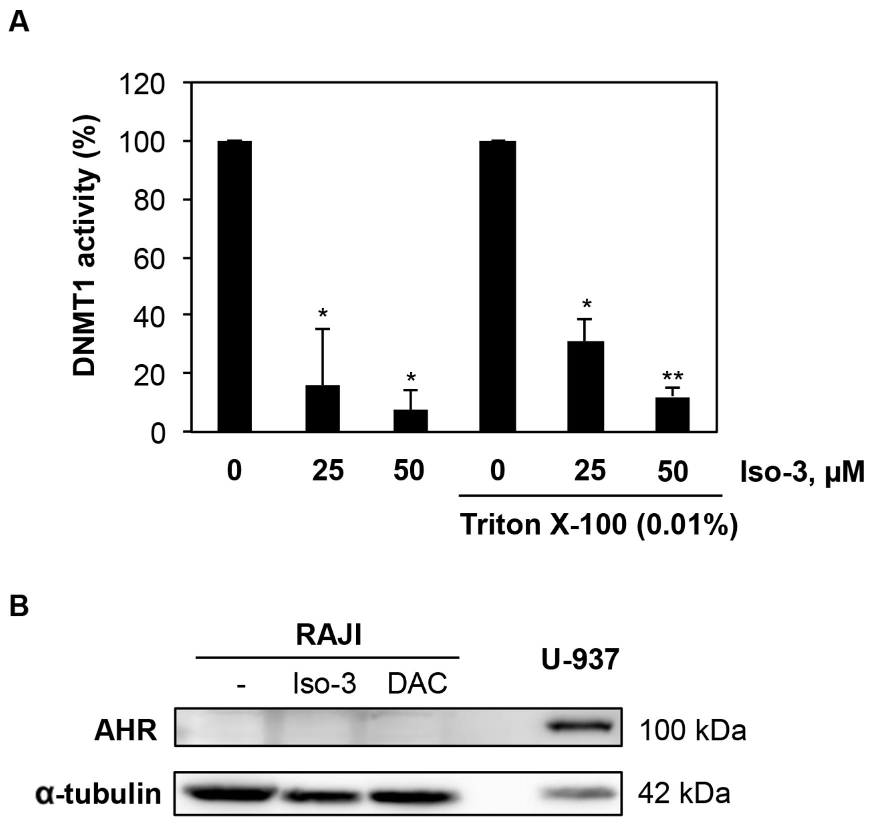

Supplementary Figure S2: (A) DNMT1 activity assay was performed in presence or absence of 0.01% TritonX-100. Histogram represents the mean ± SD of at least 3 independent experiments. (**B**) AHR protein levels were detected by Western Blot analysis upon Iso-3 (25 μM) or DAC (1 μM) treatment for 72 h in RAJI cells. U-937 cells were used as positive control for AHR expression. Blot is representative of three independent experiments.

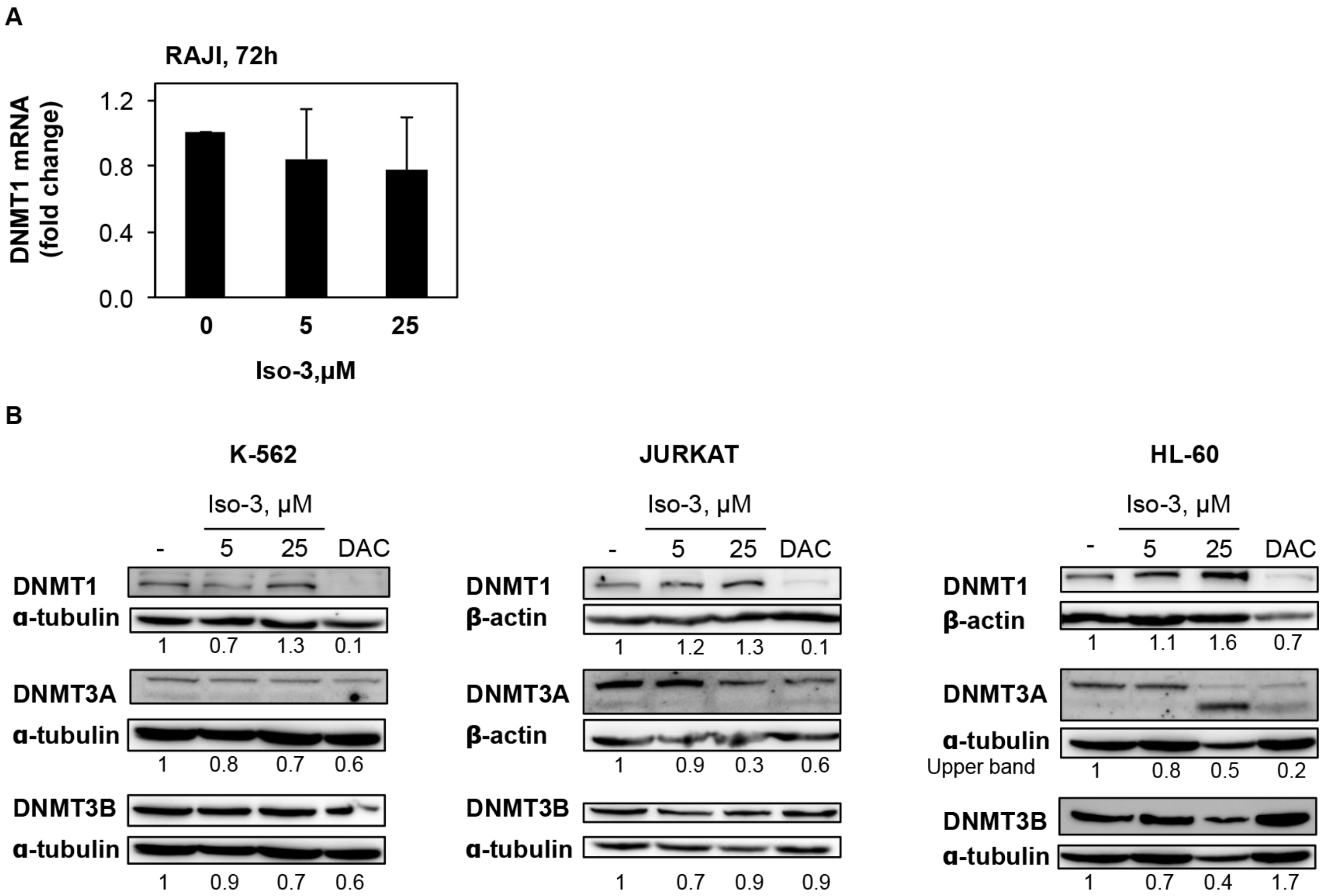

Supplementary Figure S3: (A) DNMT1 mRNA levels in RAJI cells were measured by RT-PCR after 72 h Iso-3 treatment. Histograms represent the mean ± SD of three independent experiments. (**B**) Western Blot analysis of DNMT isoforms in K-562, JURKAT and HL-60 cells after 72 h treatment with indicated Iso-3 doses or 1 µM DAC. Blots are representative of three independent experiments.

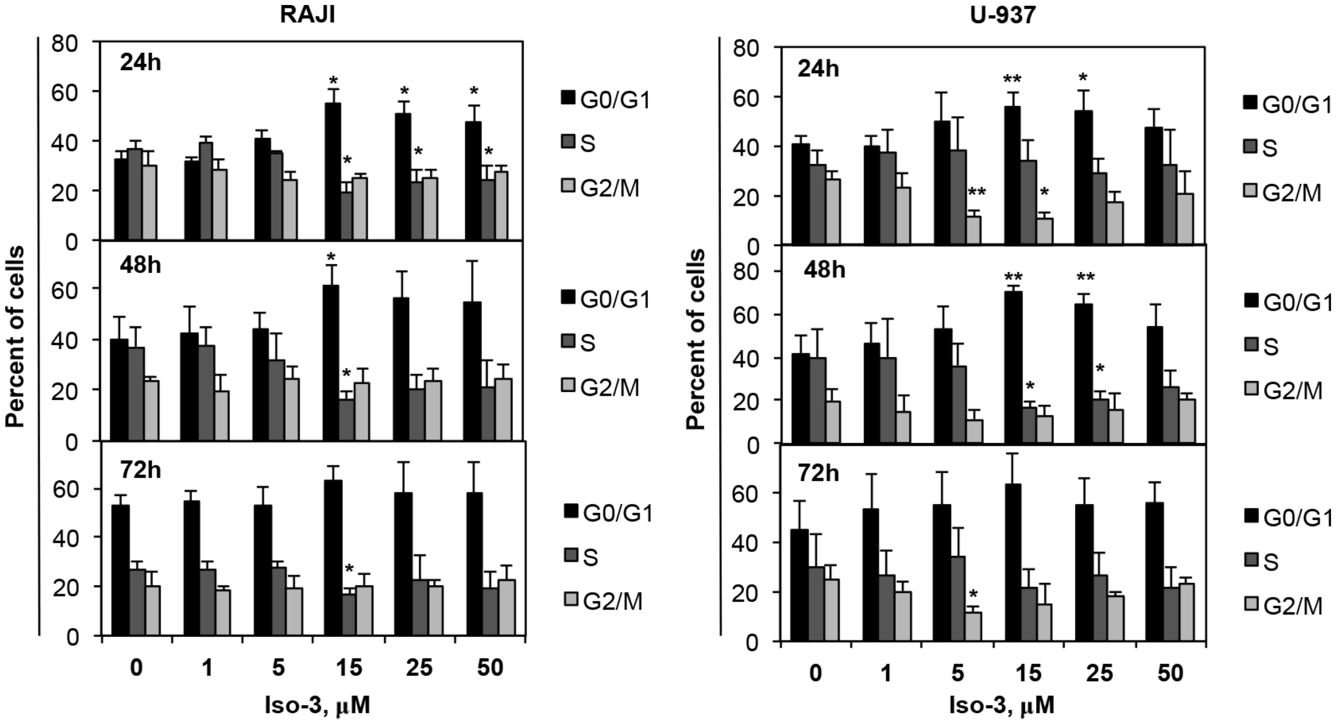

Supplementary Figure S4: Cell cycle progression analysis of RAJI and U-937 cells after 24, 48 and 72 h of Iso-3 treatment at the indicated doses. Histograms represent the mean ± SD of three independent experiments.

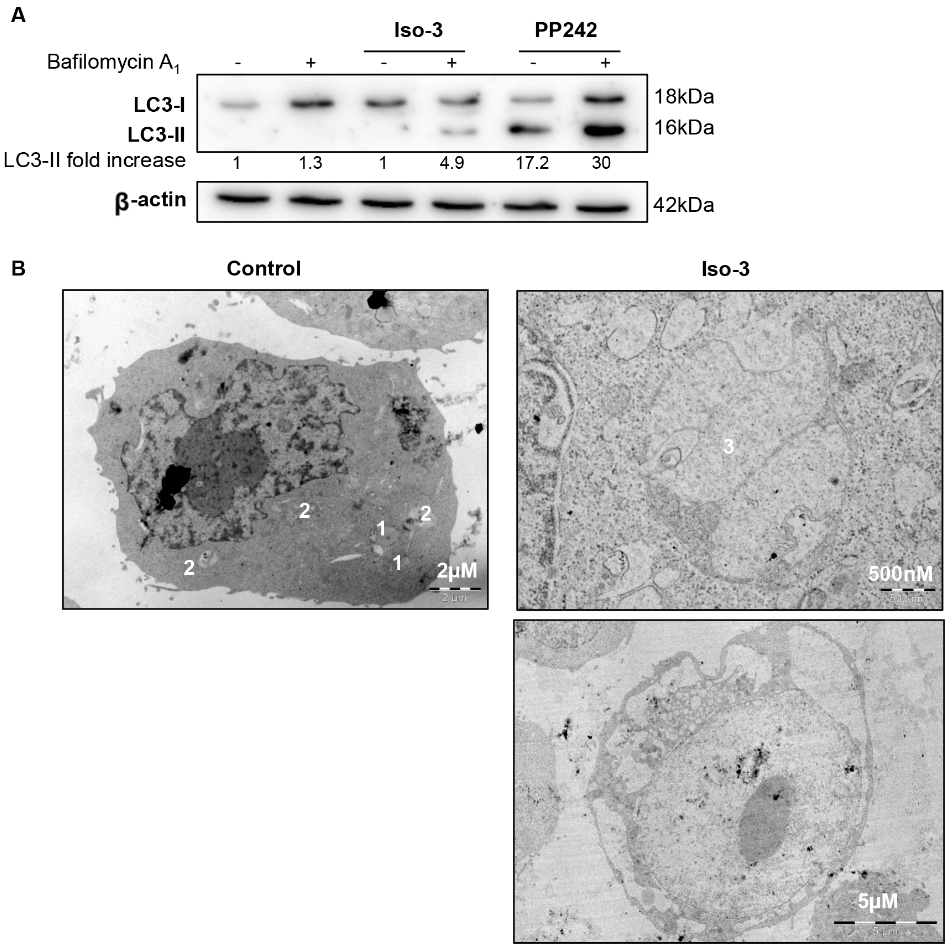

Supplementary Figure S5: (A) Western blot analysis of LC3 conversion in RAJI cells treated with 15 µM Iso-3 for 12 h. Where indicated, bafilomycin A_1 (40 nM) was added 2 h before harvesting. Cells treated 4 h with PP242 (10 µM) were used as a positive control for autophagy induction. Blot is representative of three independent experiments. (**B**) RAJI control cells exhibit low basal autophagy levels. (1) Lysosomes, (2) autolysosomes. A RAJI cell treated with 15 µM Iso-3 for 24 h shows a large autophagocytic vacuole (upper image); in extreme cases, autophagy induction culminates in autophagic cell death, evidenced by extensive vacuolization, organelle depletion while nucleus is non-pyknotic (lower image). (3) A large autophagocytic vacuole with membrane remnants.

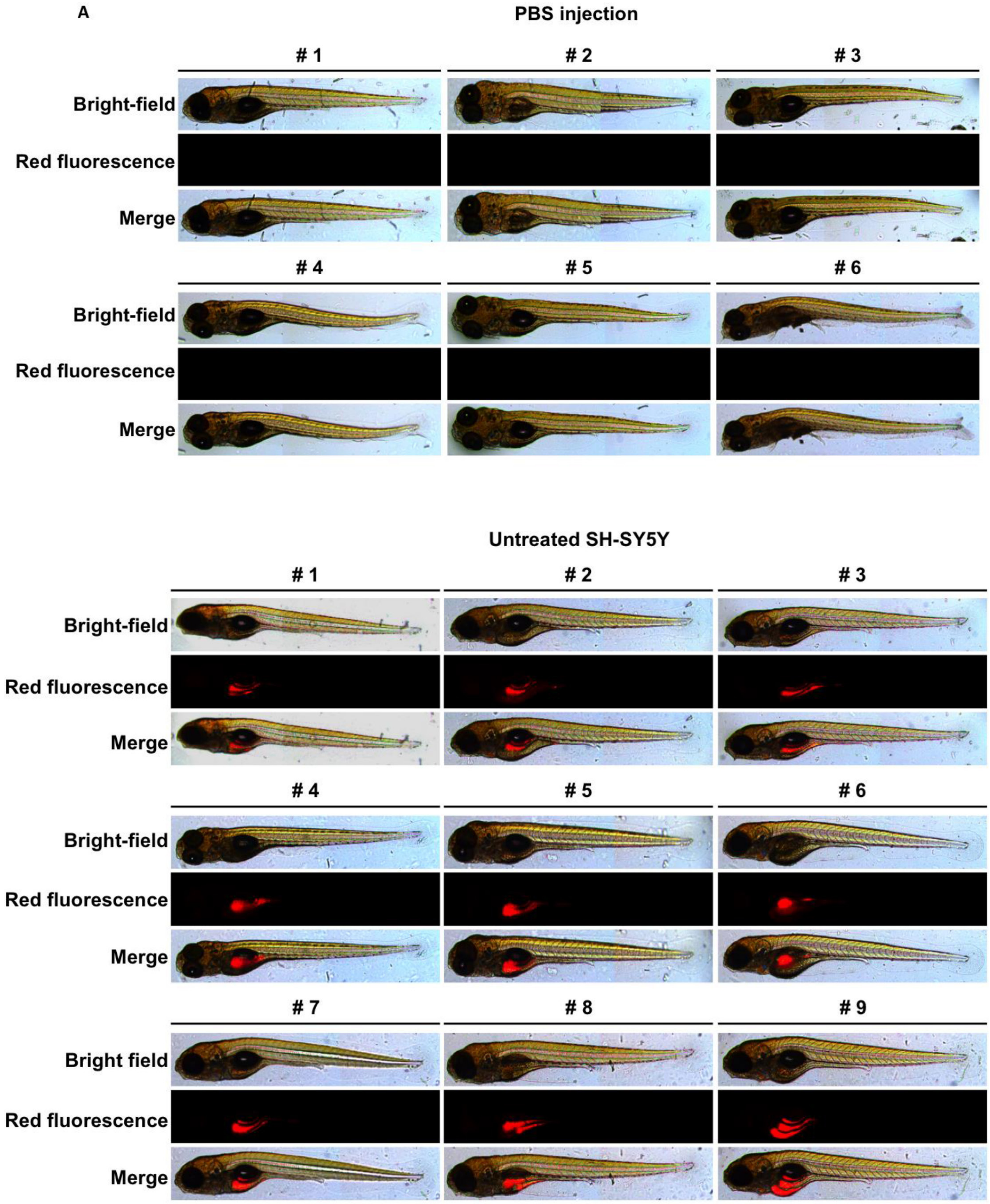
A
PBS injection
1
2
3
Bright-field
Red fluorescence
Merge
4
5
6
Bright-field
Red fluorescence
Merge
Untreated SH-SY5Y
1
2
3
Bright-field
Red fluorescence
Merge
4
5
6
Bright-field
Red fluorescence
Merge
7
8
9
Bright field
Red fluorescence
Merge

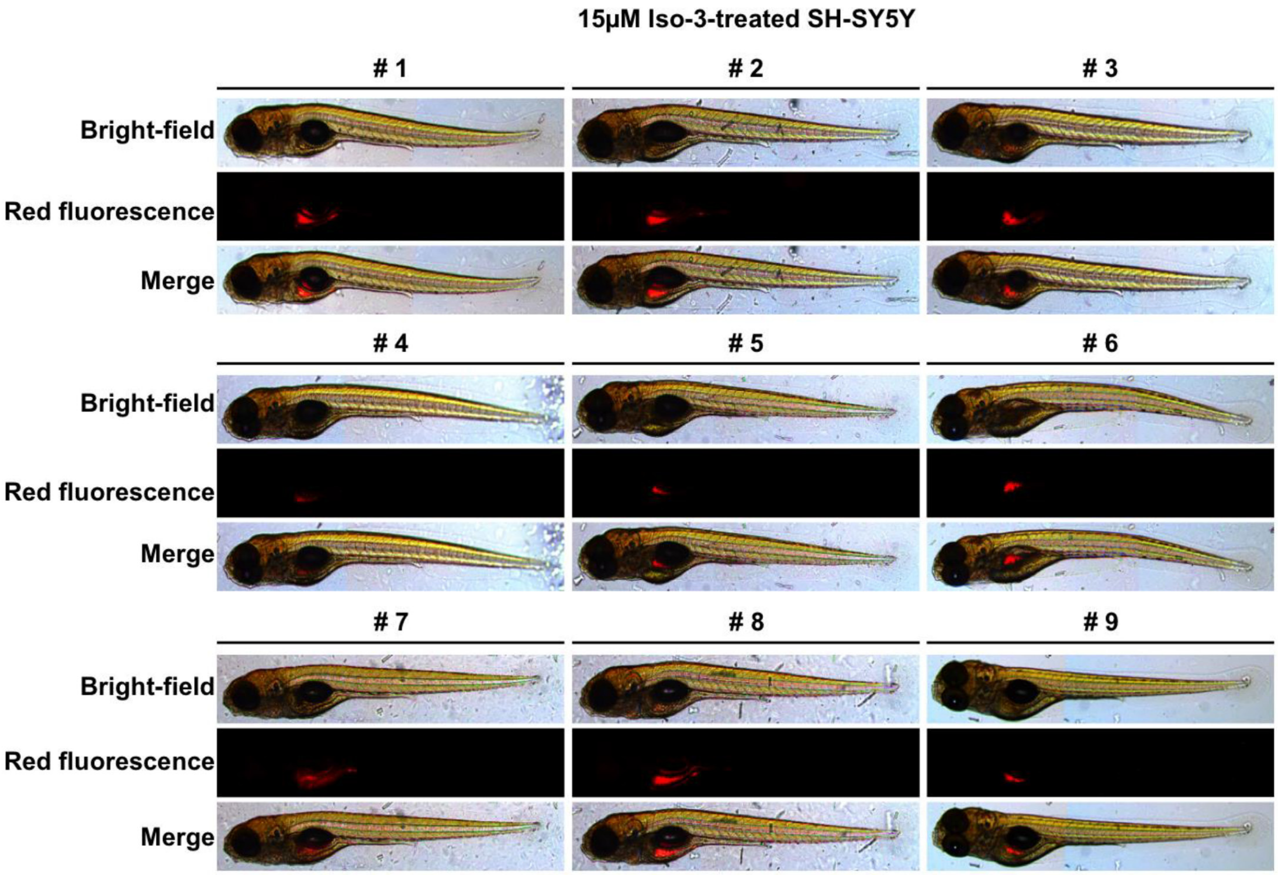

15μM Iso-3-treated SH-SY5Y
1
2
3
Bright-field
Red fluorescence
Merge
4
5
6
Bright-field
Red fluorescence
Merge
7
8
9
Bright-field
Red fluorescence
Merge

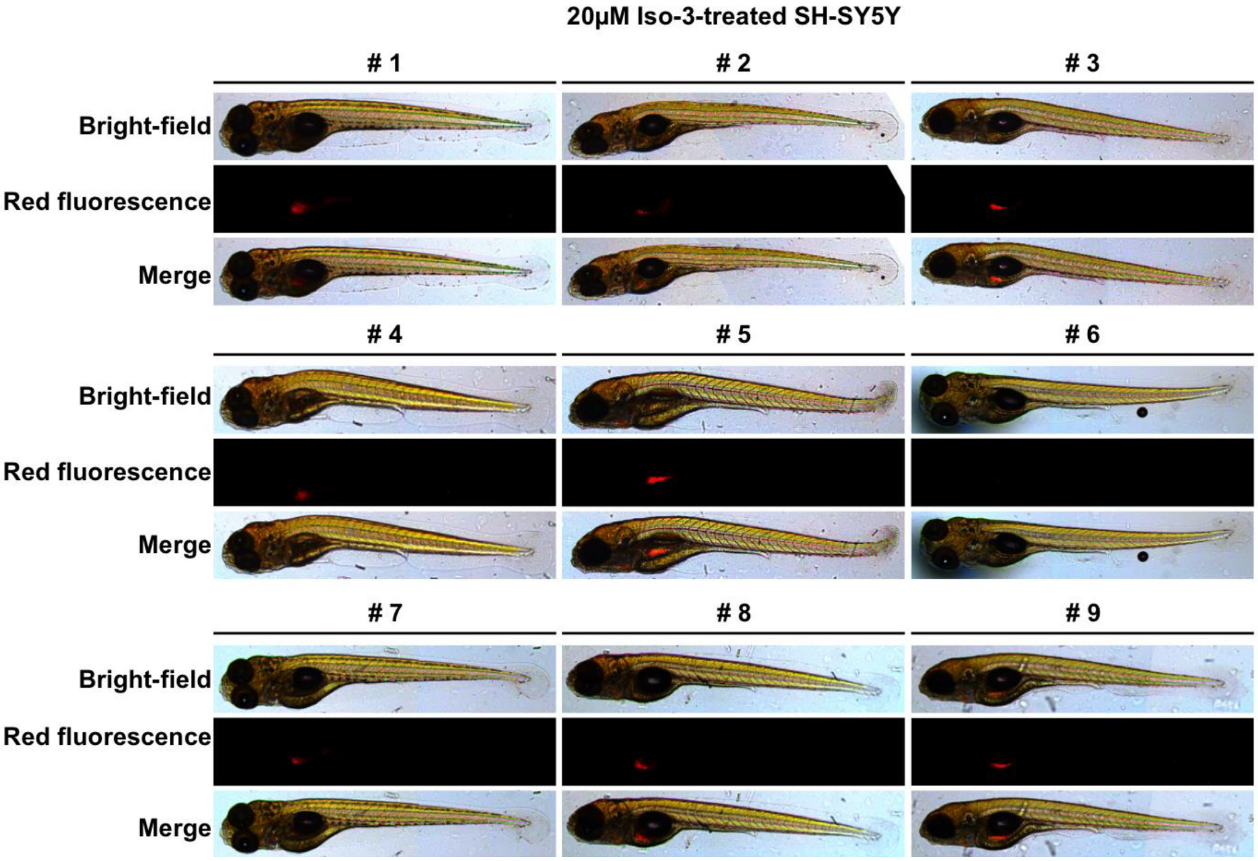

20μM Iso-3-treated SH-SY5Y
1
2
3
Bright-field
Red fluorescence
Merge
4
5
6
Bright-field
Red fluorescence
Merge
7
8
9
Bright-field
Red fluorescence
Merge

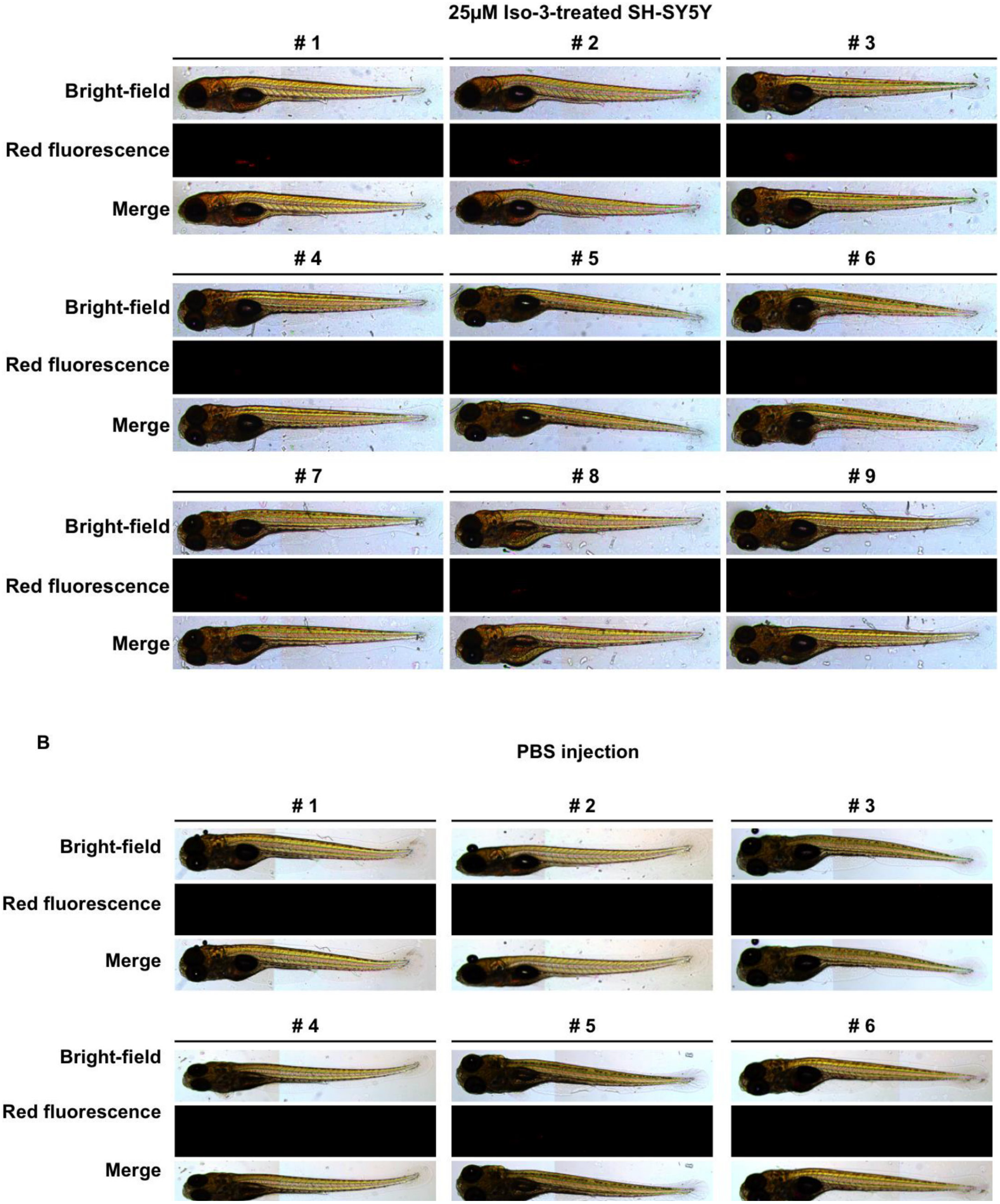

25μM Iso-3-treated SH-SY5Y
1
2
3
4
5
6
7
8
9
Bright-field
Red fluorescence
Merge
B
PBS injection
1
2
3
4
5
6
Bright-field
Red fluorescence
Merge

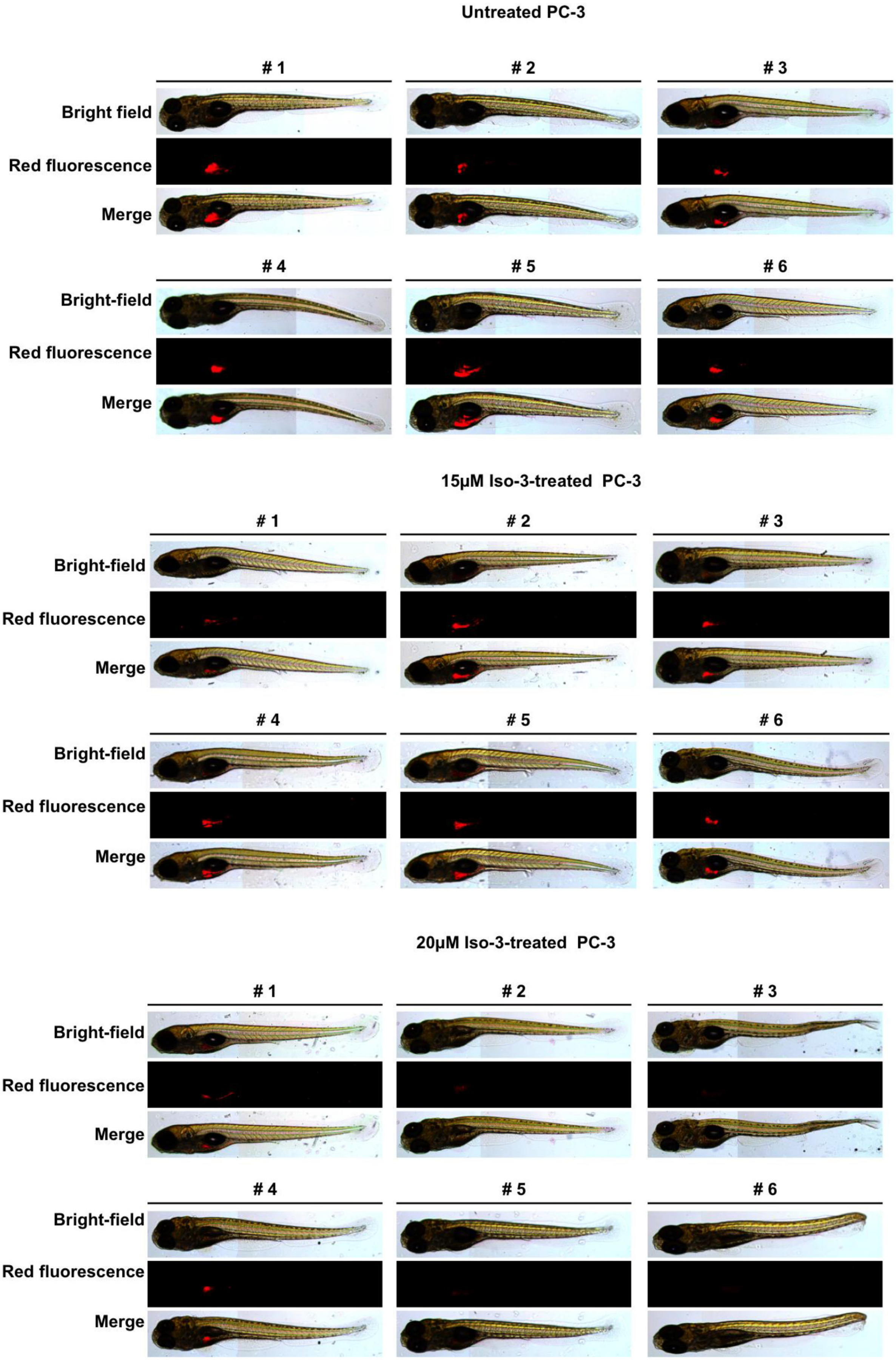
Untreated PC-3
1
2
3
Bright field
Red fluorescence
Merge
4
5
6
Bright-field
Red fluorescence
Merge
15μM Iso-3-treated PC-3
1
2
3
Bright-field
Red fluorescence
Merge
4
5
6
Bright-field
Red fluorescence
Merge
20μM Iso-3-treated PC-3
1
2
3
Bright-field
Red fluorescence
Merge
4
5
6
Bright-field
Red fluorescence
Merge

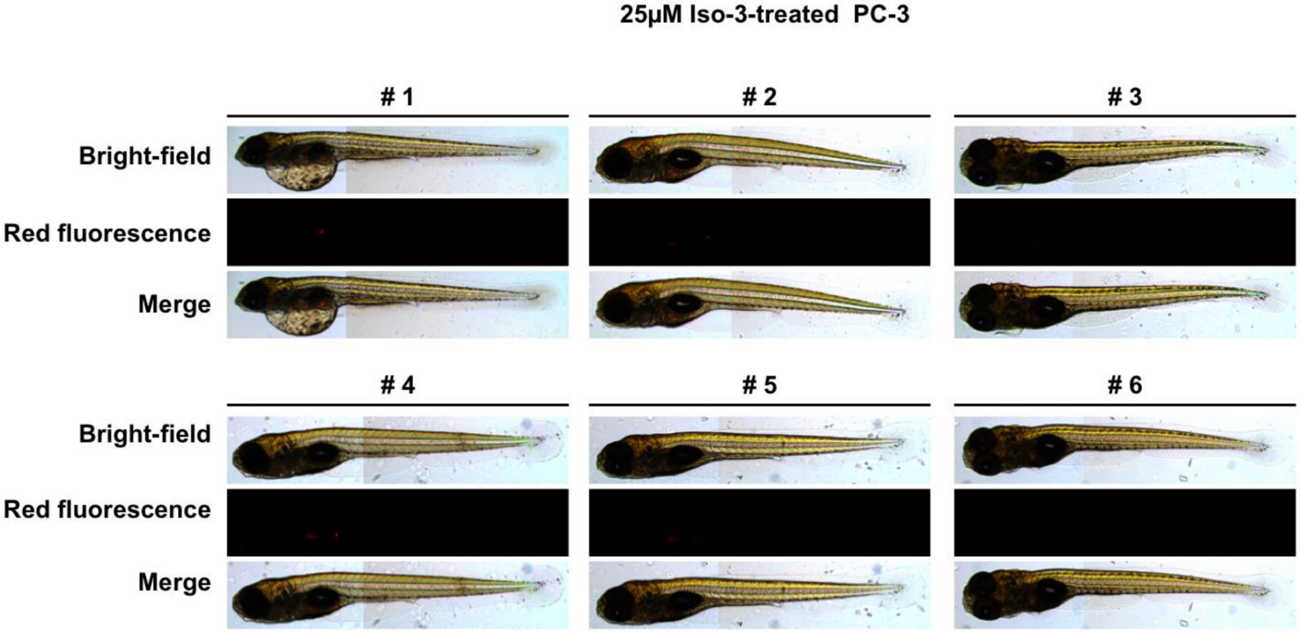

Supplementary Figure S6: Fluorescent SH-SY5Y (A) or PC-3 (B) cells were treated or not *in vitro* at different concentrations of Iso-3 for 24 h and then injected into the zebrafish yolk sac. Fluorescence was scored after 72 h. PBS injection was used as a control for injection toxicity.

Supplementary Table S1: Compounds with *in vitro* DNMT inhibitory activity

Compound	Residual total DNMT activity at 25 µM (%)	Residual DNMT1 activity at 25 µM (%)
**Aerothionin**	76.3 ± 18.1	77.9 ± 9.1
**Agelanesin A + B**	69.6 ± 10.4	Inactive*
**Alterporriol F**	72.6 ± 32.0	Inactive
**AM7b 5.3.7.6**	Not tested	66.4 ± 4.6**
**Avarone**	41.9 ± 7.0	54.9 ± 4.7
**Isofistularin-3**	73.4 ± 16.2	43.2 ± 12.4
**Mauritamide C**	78.3 ± 4.1	Not tested
**Talaromanin B**	37.0 ± 23.4	12.5 ± 2.6

*Inactive means less than 15% inhibition at 25 µM, **Residual DNMT1 activity at 50 µM.
Values represent the mean ± SD of 3 independent experiments. Structures are displayed in Supplementary Figure 1 (except for Isofistularin-3, Figure 1).
